# Genome Mining and Characterization of Two Novel *Lacticaseibacillus rhamnosus* Probiotic Candidates with Bile Salt Hydrolase Activity

**DOI:** 10.3390/biom15010086

**Published:** 2025-01-08

**Authors:** Gianluigi Agolino, Marianna Cristofolini, Amanda Vaccalluzzo, Davide Tagliazucchi, Alice Cattivelli, Alessandra Pino, Cinzia Caggia, Lisa Solieri, Cinzia Lucia Randazzo

**Affiliations:** 1 Department of Agriculture, Food and Environment, University of Catania, 95123 Catania, Italy; 2 Department of Life Science, University of Modena and Reggio Emilia, 42122 Reggio Emilia, Italy; 3 ProBioEtna SRL, Spin off of the University of Catania, 95123 Catania, Italy

**Keywords:** *Lacticaseibacillus rhamnosus*, bile salt hydrolase, bile acids deconjugation, probiotics, bile acids stress

## Abstract

Bile salt hydrolase (BSH; EC 3.5.1.24) is the microbial enzyme that catalyzes the conversion of primary bile acids (BAs) into secondary ones, promoting microbial adaptation and modulating several host’s biological functions. Probiotics with BSH activity are supposed to survive harsh intestinal conditions and exert a cholesterol-lowering effect. Here, *Lacticaseibacillus rhamnosus* strains (VB4 and VB1), isolated from the vaginal ecosystem, were submitted to a genomic survey, in vitro BSH activity, and BAs tolerance assay to unravel their probiotic potential as BAs modulators. The draft genomes of *Lcb. rhamnosus* VB4 and VB1 strains comprised 2769 and 2704 CDSs, respectively. Gene annotation revealed numerous strain-specific genes involved in metabolism and transport, as well as in DNA recombination. Each strain harbors a single *bsh* gene, encoding a C-N amide hydrolase, which conserved the essential residues required in the BSH core site. According to the results, compared to VB1, the VB4 strain tolerated better BAs stress and was more active in deconjugating BAs. However, BAs stress increased the *bsh* gene transcription in the VB1 strain but not in the VB4 strain, suggesting a partially nonlinear relationship between BSH activity and gene expression. In conclusion, despite the complexity of the BSH transcriptional system, the results support the VB4 strain as a promising BAs-deconjugating probiotic candidate.

## 1. Introduction

Bile acids (BAs) are versatile signal molecules with endocrine and paracrine functions, mainly involved in gut uptake of endogenous and dietary lipids (i.e., fatty acids, triglycerides, and cholesterol), as well as fat-soluble vitamins [1,2]. Primary BAs derive from cholesterol in the liver and consist of an amphipathic steroid ring conjugated at the C24 carboxyl group to either glycine or taurine. They reach the small intestine through the common bile duct, where a part of BAs is altered by the gut microbiota, whereas the major part is reabsorbed by hepatic cells and returns to the liver via enterohepatic circulation [1,3,4]. Conjugated BAs, released into the duodenum, are subjected to several modifications by the gut microbial community [2,5]. Bile salt hydrolase (BSH; EC 3.5.1.24) is a cysteine hydrolase belonging to the N-terminal nucleophile (Ntn) hydrolase superfamily, recognized as the crucial enzymatic mediator of gut microbiome–host interactions and BAs-associated biological processes [2,6]. This enzyme catalyzes the deconjugation of primary BAs, acting as a ‘gatekeeper’ for subsequent microbial transformations of the deconjugated forms into secondary BAs [3,7,8]. Over the years, researchers have shown that microbial BSHs shape the host’s secondary BAs pool, ultimately regulating host and microbial physiology [1,2,9,10,11,12], but the mechanisms underpinning these effects remain elusive [3,13]. BSH activity, exerted by microorganisms, is associated with positive effects in regulating lipid absorption and cholesterol metabolism, as well as in maintaining glucose body balance [1,5,7,10]. Furthermore, BAs contribute to gut microbiota homeostasis, exhibiting detergent-like antibacterial action, immunomodulatory, and anti-inflammatory effects [7]. Historically, BSH has been proposed to support the colonization and survivability of bacteria into the human intestine, thanks to their role in bile detoxification [1,13], but contrasting evidence on this action has been recently collected [12,14]. Generally, BSH activity has been listed as a desirable probiotic feature, according to World Health Organization (WHO) selection criteria [15]. BSH activity is constitutively expressed in the major gut microbial families such as *Lactobacillaceae*, *Bifidobacteriaceae*, *Enterococcaceae*, *Enterobacteriaceae*, and *Clostridiaceae* [2,8]. Among BSH-encoding bacteria, lactobacilli have been documented to strongly contribute to the majority of the total BSH activity in vivo [16] and their ability to deconjugate BAs is generally associated to the overall cholesterol-lowering effect in vivo [13]. In lactobacilli genomes the number of paralogous *bsh* genes varies in relation to species, strains, and lifestyle [16,17]. For instance, *Lactobacillus acidophilus* ATCC 4796, *Lactobacillus gasseri* ATCC 33323, and *Limosilactibacillus fermentum* MTCC 8711 encode two *bsh* genes, respectively [12,18,19], while *Lactiplanctibacillus plantarum* WCFS1 four *bsh* genes [20]. *Lactobacillaceae* BSHs are also highly variable in substrate preferences with consequences on lactobacilli survival and host colonization [12,17].

*Lacticaseibacillus rhamnosus* is a nomadic species which has been broadly reported as capable of exerting beneficial health effects. This species has a Qualified Presumption of Safety (QPS) status and is naturally present in the gastrointestinal tract, in vaginal microbiota, and in fermented food [21]. Since the description of *Lcb. rhamnosus* GG in 1989, many other *Lcb. rhamnosus* strains with beneficial properties on human health have been fully characterized and classified as probiotics [22]. Many of them have been proven to possess cholesterol-lowering activity, often associated with BSH activity and the presence of putative *bsh* genes [23,24,25,26]. However, the role of the *bsh* gene in *Lcb. rhamnosus* BSH activity has been not deeply investigated. Specifically, the *Lcb. rhamnosus* genome generally contains one single copy of the *bsh* gene [3], but recently the classification of this gene has been questioned in *Lcb. rhamnosus*. Song et al. [16] divided lactobacilli BSHs in four phylotypes and assigned *Lcb. rhamnosus* BSH to the phylotype BSH-T0, while O’Flaherty et al. [17] identified the putative BSH-encoding gene of *Lcb. rhamnosus* GG as homologous to penicillin V acylase (PVA), another member of Ntn hydrolase superfamily strongly related to BSH. 

The present study aimed to comprehensively analyze the genomes of two human-derived *Lcb. rhamnosus* candidates, previously selected for their in vitro ability to deconjugate BAs. In order to better understand how their genetic structure was related to this ability, a transcriptional study on the putative BSH-encoding gene candidates was coupled to growth assays and in vitro screening for BA conjugating activity by LC–MS/MS.

## 2. Materials and Methods

### 2.1. Reagents

Unless otherwise specified, the media were purchased from Oxoid (Basingstoke, Hampshire, UK), whereas the reagents were obtained from Sigma-Aldrich (St. Louis, MO, USA). Anaerobic systems and molecular biology reagents were bought from Thermo Fisher Scientific (Waltham, MA, USA). The BMR Genomics (Padova, Italy) supplied oligonucleotides and performed the Sanger sequencing services.

### 2.2. Bacterial Strains and Culture Conditions

*Lacticaseibacillus rhamnosus* strains, used in the present study, belonged to the microbial culture collection of ProBioEtna srl, Spin Off of the University of Catania, Italy. The strains, preserved at −80 °C in de Man, Rogosa and Sharpe (MRS) medium (pH 6.5) containing 25% (*v*/*v*) of glycerol, were routinely propagated in MRS medium, supplemented with 1.5% (*w*/*v*) agar when required, and incubated at 37 °C for 24 h.

### 2.3. Genomic Sequencing, Annotation, and Comparative Genome Analysis

The DNA extraction was carried out through the QIAcube (Qiagen, Germantown MD, USA) automated extraction system using the DNeasy^®^ UltraClean^®^ Microbial Kit (Qiagen, Germantown MD, USA), according to the manufacturer’s instructions for Gram-positive bacteria. The concentration and purity of the DNA were evaluated using a NanoDrop ND-1000 spectrophotometer (Thermo Fisher Scientific, Waltham, MA, USA). For the whole-genome sequencing, the gDNA integrity was assessed by agarose gel electrophoresis and the purity was checked by a spectrophotometer, according to an OD_260_/OD_280_ ratio of 1.8–2.0, and an OD_260_/OD_230_ ratio of 2.0–2.2 was used for the whole-genome sequencing. Synbiotec srl (Camerino, Italy) performed both the library preparation and genome sequencing. Briefly, the genomes were sequenced with the Illumina MiSeq Sequencing System, using the proprietary V3 reagents kit, producing 2 × 150 bp paired-end reads. The raw reads were trimmed with Trimmomatic version 0.39 [27] and de novo assembly was performed with Unicycler version 0.5.0 [28]. The genome quality was evaluated with the software BUSCO version 5.5.0 [29] using lactobacillales_odb10 (v2020-03-06) as the lineage dataset. The genome annotation was performed with BAKTA v. 1.7.0 [30]. Customized graphical maps of genomes were achieved through the Proksee Server version 1.1.2 [31] using the annotation file in GenBank format (gbk). The KEGG functional annotation was performed by BLASTKOALA version 3.0 [32]. The comparative genome analysis was carried out with PanExplorer v2.0 web-based tool [33]. Three reference genomes included in the analysis were chosen from experimentally validated *Lcb. rhamnosus* probiotics, such as strains Lc705 (FM179323.1), GR-1 (CP102542.1), and GG (CP031290.1).

### 2.4. Taxonomic Identification and Phylogenomics

The 16S rRNA gene was used for the initial species identification. Similarities of the 16S rRNA genes to the NCBI RefSeq database [34] were searched using the nucleotide basic local alignment search tool available at NCBI (accessed on 1 June 2024). The Muscle program [35] and the Neighbor-joining method [36] were used to align the sequences and to perform the phylogenetic tree analysis, with the bootstrap test of 1000 replicates by MEGA11 [37], respectively. The evolutionary distances in the units of the number of base substitutions per site were computed using the Kimura 2-parameter method [38]. To draw the resulting phylogenetic tree, the Interactive Tree Of Life (iTOL version 7) was used [39]. A cutoff of 98.7% 16S rRNA gene similarity was applied for the species attribution [40]. *Weizmannia coagulans*, *Bacillus subtilis*, *Bacillus vallismortis*, and *Enterococcus faecalis* were included in the analysis as outgroup species [41].

### 2.5. Safety and Genome Stability Analyses

The presence of prophages and virulence genes was detected and mapped with Phigaro v2.3.0 [42] and VirulenceFinder 2.0 [43], respectively. Antimicrobial resistance (AMR) genes were searched through CARD v4.0 [44] and ResFinder v4.3.2 [45] tools. Putative plasmids were identified using the PlasmidFinder v2.1 database (https://cge.food.dtu.dk/services/PlasmidFinder/, accessed on 1 December 2024) according to the following screening criteria: a 95% identity threshold and minimum coverage of 60% [46]. CRISPR (Clustered Regularly Interspaced Short Palindromic Repeats) arrays and their corresponding Cas proteins were pinpointed by employing CRISPRCasFinder v2.2 [47]. The presence of mobile element genes was examined using BLASTX searches against the comprehensive the mobileOG-db database v1.1.3 [48] with >90% identity and >90% coverage, respectively.

### 2.6. In Silico Analysis of Bsh Gene Candidates

The amino acid sequences of the previously characterized *bsh* genes were used as queries to search *bsh* gene candidates in genome sequences of strains VB4 and VB1 through the BLASTp tool (https://blast.ncbi.nlm.nih.gov/Blast.cgi, 2 February 2024) (Supplementary Table S2). Candidate *bsh* genes in VB4 and VB1 genomes were then aligned using the Constraint-Based Alignment Tool (COBALT) [49] with 88 Refseq amino acid sequences annotated as *bsh* or putative *bsh* proteins from lactobacilli species and 6 amino acid sequences annotated as PVA proteins and chosen according to O’Flaherty et al. [17] and Daly et al. [50]. When required, sequence alignments were visualized with Jalview v2.11.4.0 [51]. A phylogenetic tree was constructed with the Fast Minimum evolution tree method [52]. The evolutionary distance between sequences was modeled according to Grishin [53] with the maximum allowed fraction of mismatched bases set up to 0.85. The resulting tree was visualized with iTOL [39].

### 2.7. Penicillin V Susceptibility

The microdilution broth method was used for the determination of the Minimum Inhibitory Concentration (MIC) value of penicillin V (PenV), based on international methodologies ISO 10932/IDF 233 [54] for the discrimination of antibiotic susceptibility referred to lactobacilli. According to that, a bacterial suspension of each strain was standardized to 1 McFarland (3.0 × 10^8^ CFU/mL) and diluted 1:100 (final concentration of 3.0 × 10^6^ CFU/mL). The experiment was performed in 96-well plates filled with 140 µL of serial two-fold dilution of penicillin V (128–0.25 µL/mg) in double-concentrated LSM broth (90% Isosensitest and 10% MRS broth; Oxoid) and 10 µL of the diluted bacterial suspension. The plates were incubated under anaerobic conditions at 37 °C for 48 h. According to the MIC cut-off values, the tested strains were classified as resistant or susceptible to the tested antibiotic, in accordance with EFSA guidelines [55].

### 2.8. Growth Curves Assay

The growth assays were performed in 100 mL screw-top flasks (Corning, Acton, MA, USA) filled with 45 mL of MRS medium containing a 1.0% (*w*/*v*) mixture of BAs or MRS without BAs, as a control, and incubated at 37 °C under static conditions. After pre-culturing each strain in 10 mL of MRS medium, the cells were transferred to each flask at a final concentration of 1.00 × 10^5^ CFU/mL. All the experiments were carried out in triplicates. The samples were aseptically withdrawn for the measurement of absorbance at 600 nm (OD_600nm_) at least three times a day and, in the stationary growth phase, additional aliquots were taken for biochemical and gene expression analyses. The resulting growth curves were modelled according to the parametric equations available in Grofit R package [56]. A indicated the maximum cell density reached by the culture at the stationary phase of growth (expressed as OD_600nm_ values), µ was the maximum growth rate (expressed as·h^−1^), and λ the length of the latency phase (expressed in h). For the mass spectrometry analysis and gene expression analysis, aliquots were taken when the OD_600nm_ values were constant for three consecutive measurements. In detail, the samples for the mass spectrometry analysis were centrifuged at 12,000× *g* for 10 min (4 °C), and the supernatants were immediately stored at −80 °C after 0.22 µm filtration. For the gene expression analysis, a volume of cell suspensions corresponding to 2.0 × 10^8^ CFU was centrifuged at 12,000× *g* for 10 min (4 °C), and the resulting pellet was washed with DEPC-treated ddH_2_O and immediately stored at −80 °C.

### 2.9. RNA Extraction, Retro-Transcription, and Gene Expression Analysis

RNA extraction was carried out using the Zymo Direct-zol RNA MiniPrep kit (R2071, Zymo Research, Irvine, CA, USA) and applying few modifications to the manufacturer’s instructions. Briefly, after adding up to 700 µL of the Tri reagent, the mechanical lysis of cells was achieved using a Vortex Genie 2 instrument (Mo Bio Laboratories Carlsbad, CA, USA) by performing two rounds of 20 min at the highest speed alternating with 3 min on ice. The quantity of total RNA was measured spectrophotometrically using a Nanodrop Nd 1000 system (Nanodrop Technologies, Wilmington, DE, USA) and only samples determined to have A260/280 absorbance ratios between 1.8 and 2.2 were considered for further analyses. The integrity of the total RNA was evaluated by denaturing gel electrophoresis on a 0.9% (*w*/*v*) agarose gel with formaldehyde (10 mL of 10× 3-morpholinepropane sulfonic acid [MOPS] running buffer) and 18 mL of 37% formaldehyde (12 mol/L) in pH 7.0 1× MOPS running buffer (0.4 mol/L MOPS, 1 mol/L sodium acetate, and 0.01 mol/L EDTA) after the RNA treatment at 65 °C for 10 min. To remove any contamination of gDNA, 1 µg of the RNA sample was treated with dsDNase (EN0771, Thermo Fisher Scientific) (final volume 40 µL) and, thereafter, RNA was reverse transcribed to cDNA at 42 °C for 60 min with random hexamer primers using the RevertAid RT Kit (EP0441; Thermo Fisher Scientific) according to the manufacturer’s instructions.

The end-point PCR amplification of the putative *bsh* gene was carried out with a Dream Taq DNA polymerase. RT-PCR of the 16S rRNA gene was used as the positive control and carried out as previously reported [57]. RT-qPCR reactions were performed with tenfold-diluted cDNA using the PowerUp SYBR Green Master Mix (A25742; Thermo Fisher Scientific) on a QuantStudio 3 real-time PCR system (Thermo Fisher Scientific, Waltham, MA, USA). Each reaction was prepared in a 20 μL mixture containing 10 μL of the Power SYBR Green master mix, 0.3 µM of each primer with the designated final concentration, and 5 μL of diluted cDNA. The thermal conditions were as follows: 50 °C for 2 min, 95 °C for 2 min, 40 cycles at 95 °C for 15 s, and then at 60 °C for 1 min with fluorescence measurement, and the melt curve stage including 95 °C for 15 s, 60 °C for 1 min, and increasing the temperature step to 95 °C at a rate of 0.15 °C/s. All the primers used in this study are listed in Supplementary Table S3. The expression of the *bsh* gene was normalized to that of the 16S rRNA gene to yield a relative transcript level. Gene expression ratios were calculated using the software tool REST 2009 based on the efficiency-corrected method [58]. All qPCR reactions were performed in triplicates including the non-template control for each target.

### 2.10. Semi-Quantitative Analysis in UHPLC/HR-MS

An Ultra-High-Performance Liquid Chromatography High-Resolution Mass Spectrometry (UHPLC/HR-MS) analysis was carried out by applying the protocol reported in [59], with minor modifications. The UHPLC Ultimate 3000 module (Thermo Fisher Scientific, San Jose, CA, USA) was used for the chromatographic separation, whereas the tandem mass spectrometry identification and semi-quantitative analysis were carried out through a Q Exactive Hybrid Quadrupole-Orbitrap Mass Spectrometer (Thermo Fisher Scientific, San Jose, CA, USA). After appropriate dilution, 10 μL of the sample were injected in the UHPLC system loaded with a C18 column (Acquity UPLC HSS C18 Reversed phase, 2.1 × 100 mm, 1.8 µm particle size, Waters, Milan, Italy). The used mobile phases were water with 0.1% formic acid (mobile phase A) and acetonitrile with 0.1% formic acid (mobile phase B). The gradient began with 58% of B, and then linearly increased to 75% of B in 10 min. Next, the percentage of mobile phase B was brought to 98% in 1 min and kept for a further 6 min before coming back to the initial conditions. The flow rate was fixed at 0.3 mL/min and the column temperature was maintained at 45 °C. The negative electrospray conditions were as indicated below: capillary voltage, 2.7 kV; capillary temperature, 320 °C; sheath gas, 40; and auxiliary gas, 30. The MS parameters were resolution, 70,000; AGC target, 3 × 10^6^; maximum IT, 247 ms; and scan range, 100 to 1500 *m*/*z*. MS/MS parameters were as follows: resolution, 17,500; AGC target, 5 × 10^5^; maximum IT, 120 ms; and isolation window, 1 *m*/*z*. 

The analyzed samples were MRS medium containing 1.0% (*w*/*v*) BAs mixture inoculated with the selected strains and incubated as reported in the Section 2.8. To determine the percentage of the decrease in BAs, a standard solution was prepared containing the mixture of BAs dissolved in MRS at the same concentration of 1.0% (*w*/*v*) as for the inoculated samples.

The relative amount of BAs was determined by integrating the area under the peak (AUP). AUP values were quantified from the extracted ion chromatograms (EIC) calculated for each mass-to-charge ratio of compound (tolerance ± 5 ppm) using the Genesis algorithm function in the Thermo Xcalibur Quantitative Browser.

The percentage of decrease for each BA, namely taurocholate (TCA), taurodeoxycholate (TDCA), taurochenodeoxycholate (TCDCA), glycocholate (GCA), glycodeoxycholate (GDCA), and glycochenodeoxycholate (GCDCA), was calculated as follows:

% decrease = 100 − [100 × (AUP of BA in inoculated sample/AUP of BA in standard solution)]

### 2.11. Statistical Analysis

All the analyses were carried out in triplicates and the data are reported as the mean ± SD. GraphPad Prism v.8.00 was used to generate graphs and to perform statistical analysis. *p*-values were calculated using two-sided Student’s *t*-tests, unless stated otherwise. Statistical significance was considered at *p* < 0.05 and the levels of significance were represented as * *p* ≤ 0.05, ** *p* ≤ 0.01, *** *p* ≤ 0.001, and **** *p* ≤ 0.0001.

## 3. Results

### 3.1. Genome Sequencing

**Figure 1:**
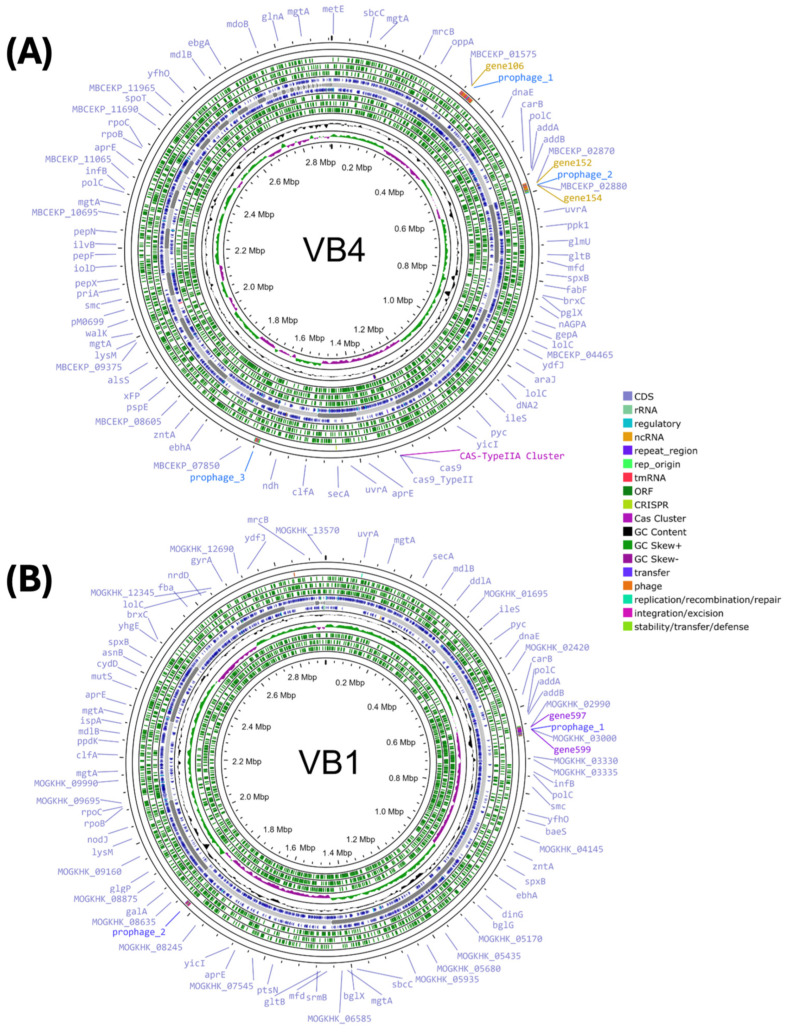
Circular graphical representations of the *Lacticaseibacillus rhamnosus* strains VB4 (**A**) and VB1 (**B**) contigs obtained using Proksee (https://proksee.ca; accessed on 13 January 2024). The black central circle shows the scale expressed in megabases. Moving inward, the two outer violet circles show forward- and reverse-strand CDSs, respectively. Some genes are shown on the outer violet circle with the Proksee’s default. In CDSs circles, tRNAs are shown as orange arrows, rRNAs are represented as light blue arrows, tmRNA is displayed as a red arrow, and CRISPR sequences are reported as light green arrows adjacent to each other. The next circle shows the GC content and GC skew as dark blue and dark green and pink, respectively. The represented genomic order of contigs is arbitrary.

Strains VB4 and VB1 were characterized at the genome level. The reads assembly resulted in 70 contigs, corresponding to a total length of 2,926,936 bp, and in 19 contigs, corresponding to a total of 2,917,389 bp, respectively, for VB4 and VB1 strains. The GC contents were 46.63% and 46.65% for strains VB4 and VB1, respectively, and a clear definition of positive and negative strands were obtained in both cases (Figure 1A,B). The genomic features of strains VB4 and VB1 are summarized in Supplementary Table S4.

### 3.2. Strain Identification

**Figure 2:**
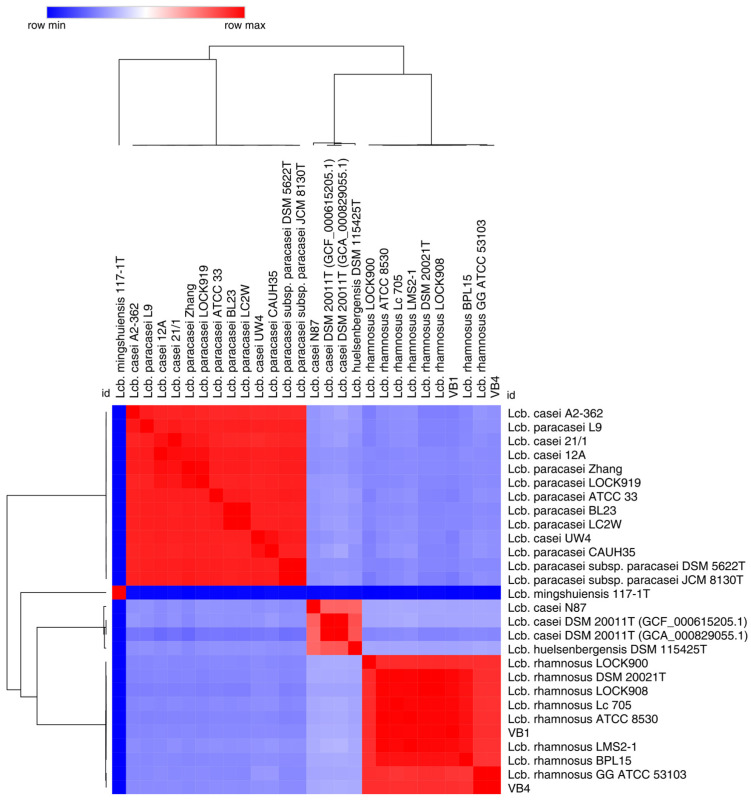
Heatmap of the average nucleotide identity (ANI) values of 28 *Lacticaseibacillus* strains. The colored squares designate the strain relatedness based on their ANI values (red color > the threshold value of 95%; dark and light blue < the threshold values of 95%).

The phylogenetic analysis of 16S rRNA gene sequences showed that strains VB4 and VB1 formed a monophyletic group with reference sequences of *Lcb. rhamnosus* NBRC 3425 and JCM 1136^T^ (Supplementary Figure S1). Accordingly, the calculation of average nucleotide identity (ANI) values showed that strains VB4 and VB1 obtained ANIb values of 97.16% and 99.74% with *Lcb. rhamnosus* DSM 20021^T^, both above the threshold for species allocation (95%) (Figure 2). The TYGS phylogenetic predictions further supported the attribution to the species *Lcb. rhamnosus* based on dDDH values (Supplementary Table S5). Based on ANIb and dDDH values, strain VB4 appeared more related to *Lcb. rhamnosus* GG than strain VB1.

### 3.3. Annotation and Comparative Genome Analysis

Genome annotation predicted a total of 2824 and 2760 genes in VB4 and VB1 genome assemblies, respectively, including 2769 (VB4) and 2704 (VB1) CDS, 45 (VB4) and 46 (VB1) tRNA, 4 (VB4) and 2 (VB1) rRNA, and 1 (VB4) and 1 (VB1) tmRNA, respectively (Supplementary Table S4).

For the VB4 genome, the KEGG functional annotation by BLASTKOALA assigned approximately half of the genes (51.0%, 1413 genes) into 23 different functional categories, mostly related to carbohydrate metabolism (234, 16.57%), protein families: genetic information processing (13.31%), and protein families: signaling and cellular processes (9.91%), among others (Supplementary Figure S2A). The KEGG functional annotation of the VB1 genome by BLASTKOALA revealed that 55.5% of the genes were assigned to 23 functional categories with slightly different proportions compared to VB4 (Supplementary Figure S2B).

**Figure 3:**
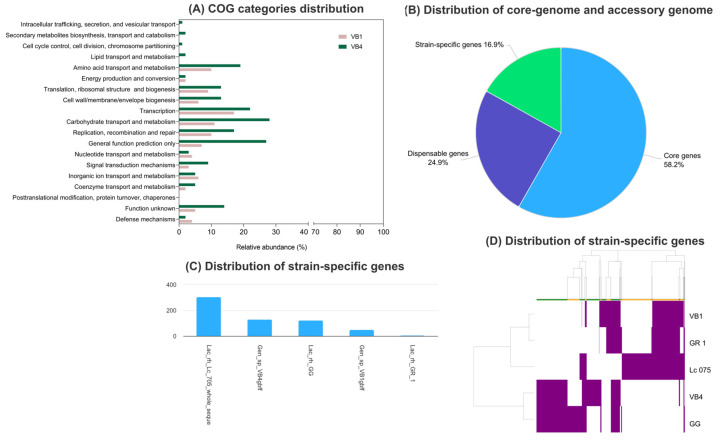
Pan-genome analysis of *Lacticaseibacillus rhamnosus* strains VB4 and VB1. The strains were compared to *Lcb. rhamnosus* strains Lc705 (ASM2652), GR-1 (ASM2466559), and GG (ASM2847508) using PanExplorer (last accessed on 15 May 2024). (**A**) Distribution of COG functional groups in VB4 and VB1 genomes. (**B**) Core-genes proportion and strain-specific genes; (**C**) distribution of strain-specific genes according to the *Lcb. rhamnosus* pan-genome analysis; and (**D**) distance tree generated by hierarchical clustering from presence/absence binary matrix of accessory gene clusters among the members of *Lcb. rhamnosus* dataset. Genes are colored if present in the genome.

Based on the clusters of orthologous groups (COG), the VB4 and VB1 strains differed in distribution of clusters in COG categories (Figure 3A). To identify strain-specific genes of *Lcb. rhamnosus* VB4 and VB1, we compared them to the genome sequences of *Lcb. rhamnosus* GG, *Lcb. rhamnosus* GR-1, and *Lcb. rhmanosus* La075. The pan-genome consisted of 3653 COG, containing a core-gene of 58.3% and a strain-specific gene pool of 16.9%. Non-necessary genes, defined as lacking in at least one of the strains, accounted for 24.9% (Figure 3B) and, together with the strain-specific genes, constituted the accessory genome. The distribution of strain-specific clusters showed that VB4 and VB1 differed in the number of singletons. Specifically, 130 genes were uniquely located in the genome of the *Lcb. rhamnosus* VB4, whereas 50 genes were uniquely assigned in the *Lcb. rhamnosus* VB1 genome (Figure 3C). The sharing pattern of accessory COG corroborated the relationships established among the *Lcb. rhamnosus* strains through the ANI analysis and supported that strain VB4 was more related to *Lcb. rhamnosus* GG, and strain VB1 to *Lcb. rhamnosus* GR-1 (Figure 3D).

**Figure 4:**
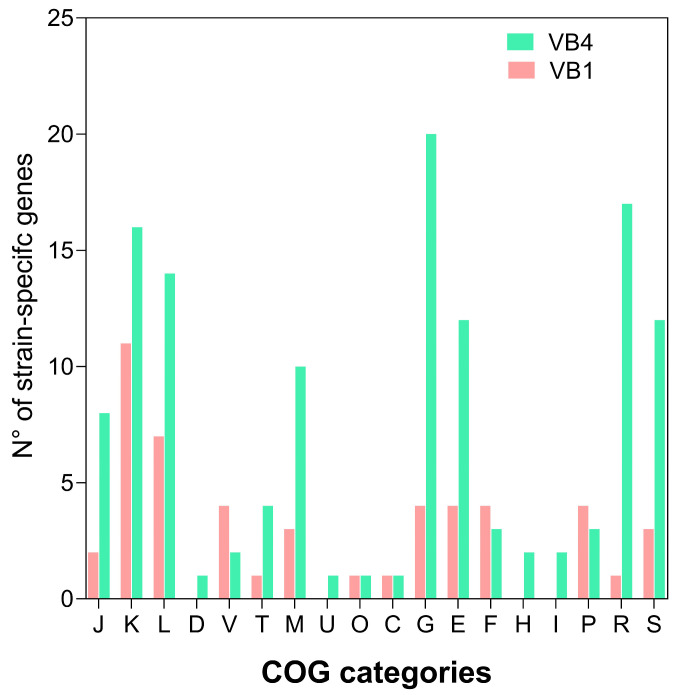
Number of unique genes in *Lacticaseibacillus rhamnosus* strains VB4 and VB1 assigned in COG functional categories. COGs of the *Lcb. rhamnosus* VB4 strain are represented as green bars whereas COGs of the *Lcb. rhamnosus* VB1 strain are displayed as pink bars. COG categories are abbreviated as follows: J; translation, ribosomal structure, and biogenesis; K, transcription; L, replication, recombination, and repair; D, cell cycle control, cell division, and chromosome partitioning; V, defense mechanisms; T, signal transduction mechanisms; M, cell wall/membrane/envelop biogenesis; U, intracellular trafficking and vesicular transport; O, post-translational modification, protein turnover, and chaperones; C, energy production and conversion; G, carbohydrate transport and metabolism; E, amino acid transport and metabolism; F, nucleotide transport and metabolism; H, coenzyme transport and metabolism; I, lipid transport and metabolism; P, inorganic ion transport and metabolism; R, general functional prediction only; and S, function unknown.

The functional analysis of strain-specific genes pointed out that three main COG categories were abundant in VB4 strain-specific genes, namely carbohydrates metabolism and transport (20), amino acid transport and metabolism (12), and cell wall/membrane/envelope biogenesis (10), but poorly represented in VB1 genome. In VB1 genome strain-specific genes mainly belonged to the categories of transcription (11) and replication, recombination, and repair (7) (Figure 4).

### 3.4. Safety Assessment

According to EFSA, the genomes of both VB4 and VB1 strains were checked for the presence of AMR genes by using two independent and maintained databases [60]. Both CARD and ResFinder analyses did not reveal AMR genes in any genome, indicating that *Lcb. rhamnosus* VB4 and VB1 can be considered safe in relation to the potential dissemination of AMR genes. The VirulenceFinder webserver did not render results for any of the strains, while PathogenFinder showed probabilities of being a human pathogen of 0.097 and 0.098 (above 1) for *Lcb. rhamnosus* VB4 and VB1, respectively. These results are in accordance with the QPS status of *Lcb. rhamnosus.*

### 3.5. Genome Stability

**Figure 5:**
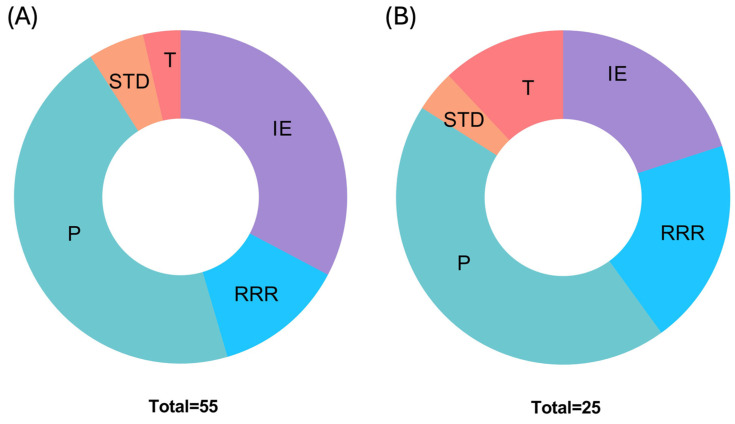
Distribution of mobile genetic elements in *Lacticaseibacillus rhamnosus* VB4 (**A**) and VB1 (**B**) genomes. Major categories considered were IE, integration/excision; RRR, replication/recombination/repair; P, phage; STD, stability/transfer/defense; and T, transfer.

The presence of plasmid replication initiation proteins in *Lcb. rhamnosus* VB4 and VB1 strains was not revealed by the PlasmidFinder (v2.1), suggesting that both strains do not possess any plasmids. The Phigaro pipeline was used to detect the prophage sequences in the genomes of *Lcb. rhamnosus* VB4 (Supplementary Figure S3A) and VB1 (Supplementary Figure S3B). Three and two prophage regions were detected in VB4 and VB1 genomes, respectively. In both strains, putative prophage regions belonged to the *Siphoviridae* family (Table 1). In the VB4 genome, contig 2 harbored a 33.6 kb long prophage region encoding 41 prophage proteins (Supplementary Figure S3A). This region was 100% identical (83% query coverage) to *Caudoviricetes* sp. isolate cttX04 (BK021713.1). In the VB1 genome, the longest prophage region (23.8 kb) was located on contig 1, contained 19 phage proteins, and was 99.85% (query coverage 52%) identical to the previously described *Lcb. rhamnosus* Lc-Nu-like prophage (AY780364.1) (Supplementary Figure S3B). The genomes of both VB4 and VB1 strains were checked to delineate regions associated with mobile elements by using the mobileOG-db plugin within Proksee. The analysis confirmed that VB4 has more regions related to mobile elements than VB1, especially integrases and phage-related genes (Figure 5).

The analysis of CRISPR sequences with CRISPRCasFinder indicated that contig 9 of the VB4 genome contains a complete CRISPR–Cas system of type II-A/LsaI1, consisting of four *cas* genes (*cas1*, *cas2*, *cas9*, and *csn2*) and one 1553 bp long CRISPR array containing 23 spacers (Supplementary Tables S6 and S7). BLASTn searches compared all 23 spacers against the phage and plasmid NCBI databases and revealed full or partial matching with several *Lcb. rhamnosus*. Another CRISPR region was detected on contig 10 of the VB4 genome assembly, but without any *cas* genes in the surrounding region. In contrast, the VB1 genome encoded a Cas3 protein on contig 9, typical for a CRISPR-Cas system type I [61] (Supplementary Table S8). However, no CRISPR regions were detected near to this ORF, while the only detected CRISPR region was located on contig 1 (coordinates 88741..88886) upstream to the only transposase gene found in the VB1 genome (100% identical to ISLca2 from *Lcb. casei* BL23) (Supplementary Table S9).

### 3.6. Identification and Phylogenetic Analysis of Bsh Gene Candidates

**Figure 6:**
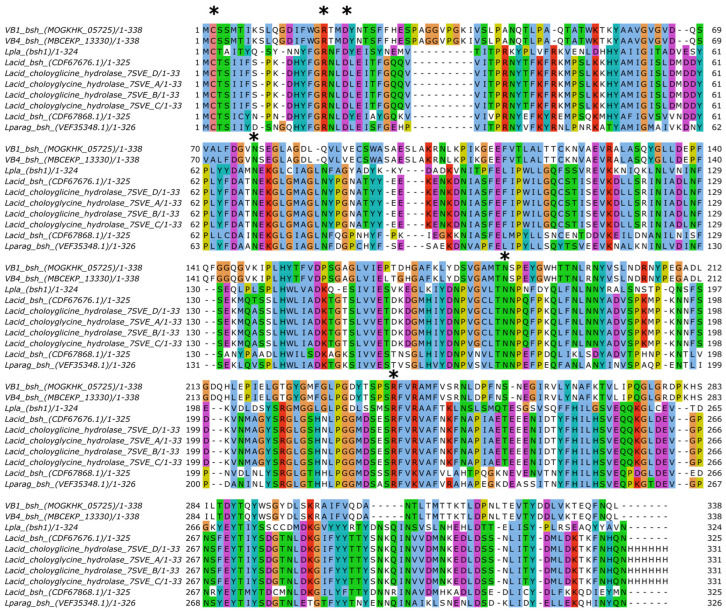
Multiple amino acid sequence alignment of 10 BSHs proteins selected from Supplementary Table S1. Asterisks indicate active sites, according to [10].

In this work, the presence of BSH enzyme-encoding genes in VB4 and VB1 genomes was investigated using the BLASTP tool (https://blast.ncbi.nlm.nih.gov/Blast.cgi; accessed on 2 February 2024) and amino acid sequences of previously characterized *bsh* genes as queries (Supplementary Table S2). We found a putative *bsh* gene on contigs 37 and 2 of VB4 and VB1 genome assemblies, respectively. In both strains, the genes were 1017 bp long and encoded two 338 aa long proteins which differed from each other for two aa substitutions, P167L and D169G, respectively. The predicted proteins were members of the choloylglycine hydrolase family and were annotated as linear C-N amide hydrolases (Pfam PF02275) (*E*-value: 4.5 × 10^−48^). In addition, they contained residues Cys2, Arg18, Asp21, Asn82, Asn175, and Arg228, which are close in 3D structure, concurring to form the central active site of the BSH enzyme, as shown in Figure 6 [10].

**Figure 7:**
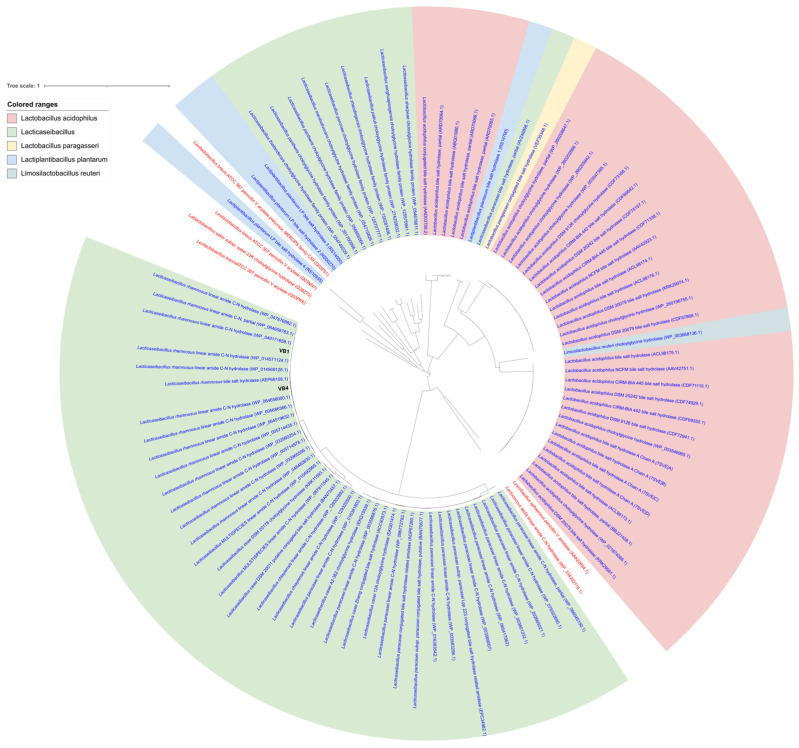
Phylogenetic tree of BSH and PVA proteins. The alignments of 96 bacterial proteins were performed with NCBI COBALT [53,56]. The resulting phylogeny was visualized as a phylogenetic tree using iTOL [39]. Red labels indicate PVA proteins.

The putative BSH proteins of VB4 and VB1 strains were aligned with 88 representative BSH proteins from 18 lactobacilli species and 6 representative PVA proteins through the COBALT alignment tool. The resulting COBALT alignment was used to calculate a phylogenetic tree. As shown in Figure 7, 39 BSH proteins from *Lcb. rhamnosus* (including VB4 and VB1) and the closest *Lacticaseibacillus* relatives (namely *Lacticaseibacillus paracasei*, *Lacticaseibacillus casei*, *Lacticaseibacillus zeae*, and *Lacticaseibacillus chyaiensis*) formed a congruent cluster together with the BSH proteins of *L. acidophilus*, *L. plantarum* (locus tag LP_RS14790), *Limosilactibalcillus reuteri*, and *Lactobacillus paragasseri*. In detail, within this cluster, putative BSH proteins clustered in three minor clusters, corresponding to *Lcb. paracasei*, *Lcb. casei* (including the closest relatives *Lcb. zeae* and *Lcb. chayiensis*), and *Lcb. rhamnosus*. BSH proteins from *Lcb. paracasei*, *Lcb. casei*, and *Lcb. rhamnosus* were also related to the PVA proteins from *Lactococcus lactis* and *Lysinibacillus sphaericus*.

### 3.7. Penicillin V Susceptibility Results

In order to exclude that predicted BSH proteins in VB4 and VB1 strains can confer PenV tolerance due to a BSH/PVA bifunctional role, the MIC assay was carried out. No visible growth was revealed by testing different PenV dilutions. VB4 and VB1 showed MIC values of 1 µg/mL and 0.5 µg/mL, respectively, and therefore, according to the EFSA breakpoint (4 µg/mL) [59], they can be considered sensitive to PenV.

### 3.8. Growth Curves, Bile Salt Deconjugation Activity, and Bsh Gene Expression Analysis in Presence of BAs Mixture

**Figure 8:**
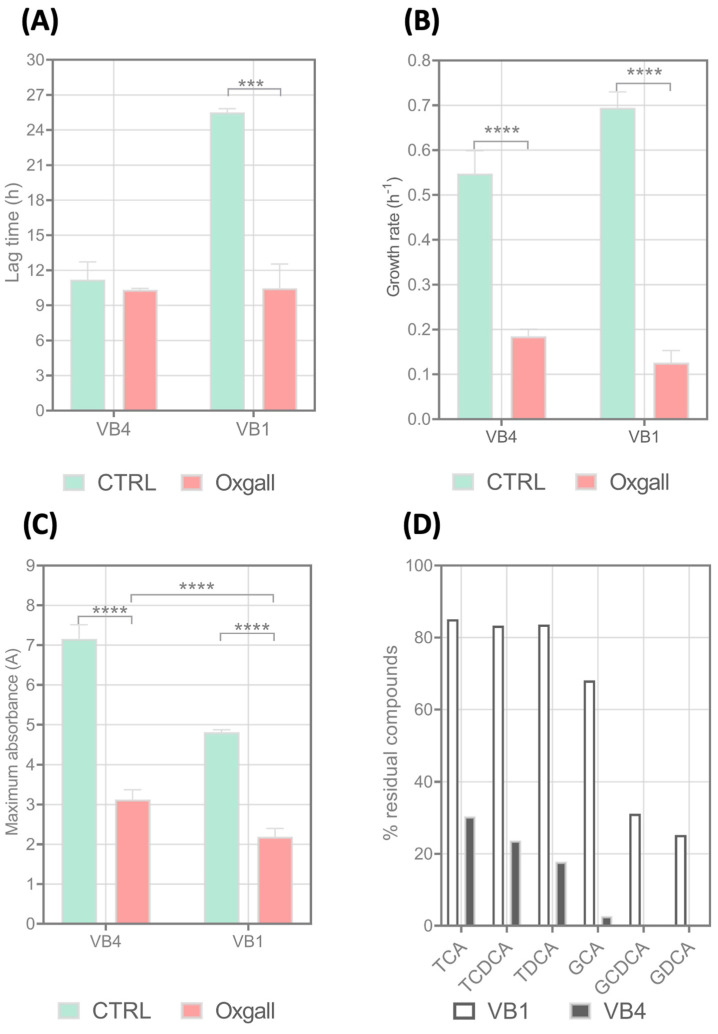
Growth and deconjugation ability of *Lacticaseibacillus rhamnosus* VB4 and VB1 strains in the presence of a BAs mixture (Oxgall). Kinetic parameters lag phase (λ, expressed in h) (**A**), maximum growth rate (µ, expressed as h^−1^) (**B**), and maximum cell density (A, expressed as OD_600 nm_) (**C**) were computed in MRS and MRS supplemented with BAs 1% (*w*/*v*) by Grofit package (version 1.1.1-1). Residual unconjugated percentages (%) of GCA, GDCA, GCDCA, TCA, TDCA, and TCDCA were estimated in supernatants collected during late stationary phase (**D**). Significant differences were calculated with two-way ANOVA and indicated with asterisks, as follows: ***, *p* ≤ 0.001; ****, *p* ≤ 0.0001 (two-way ANOVA). Plotted with GraphPad Prism v.8.00 software (San Diego, CA, USA). Abbreviations: GCA, glycocholic acid; GDCA, glycodeoxycholic acid; GCDCA, glyco-cheno-deoxycholic acid; TCA, taurocholic acid; TDCA, taurodeoxycholic acid; and TCDCA, tauro-cheno-deoxycholic acid.

Results of growth curves, in both MRS media with and without 1.0% of BAs, were reported in Supplementary Figure S4. According to that, VB4 and VB1 strains differed in growth parameters under the control condition and differently responded to BAs stress (Figure 8). Under the control condition, the VB1 strain exhibited a long lag phase which significantly decreased in response to the BAs mixture (*p* < 0.05) (Figure 8A). In contrast, the BAs mixture did not affect the lag phase of strain VB4 (*p* > 0.05). As expected, both strains significantly reduced the µ_max_ and A values in the presence of the BAs mixture compared to the control condition. In addition, the VB4 strain reduced the growth rate and maximum culture density at a lesser extent than VB1 (*p* < 0.05) (Figure 8B,C).

We examined the BAs deconjugation activity and *bsh* gene expression in VB4 and VB1 cells grown in the presence of a BAs mixture. Concerning BAs deconjugation activity, a UHPLC/HR-MS analysis of supernatants collected at the stationary phase was used to determine the BAs profiles and calculate the percentage of residual unconjugated TCA, TDCA, TCDCA, GCA, GCDA, and GCDCA. As reported in Figure 8D, despite the high similarity of putative BSH proteins, strains VB4 and VB1 exhibited significant differences in the ability to deconjugate BAs. In particular, the VB1 strain was active in deconjugating GCDCA and GDCA, leaving only 31.1% and 25.2% of conjugated residual compounds in the medium, respectively. However, the VB1 strain was poorly active in deconjugating GCA (residual percentage of 68.1%) and almost completely inactive in deconjugating the tauro-conjugated BAs. Differently, strain VB4 was found to be able to deconjugate TCA, TDCA, and TCDCA at high extent and was more active than VB1 in deconjugating glyco-conjugated BAs, such as GCDCA, GDCA, and GCA. 

**Figure 9:**
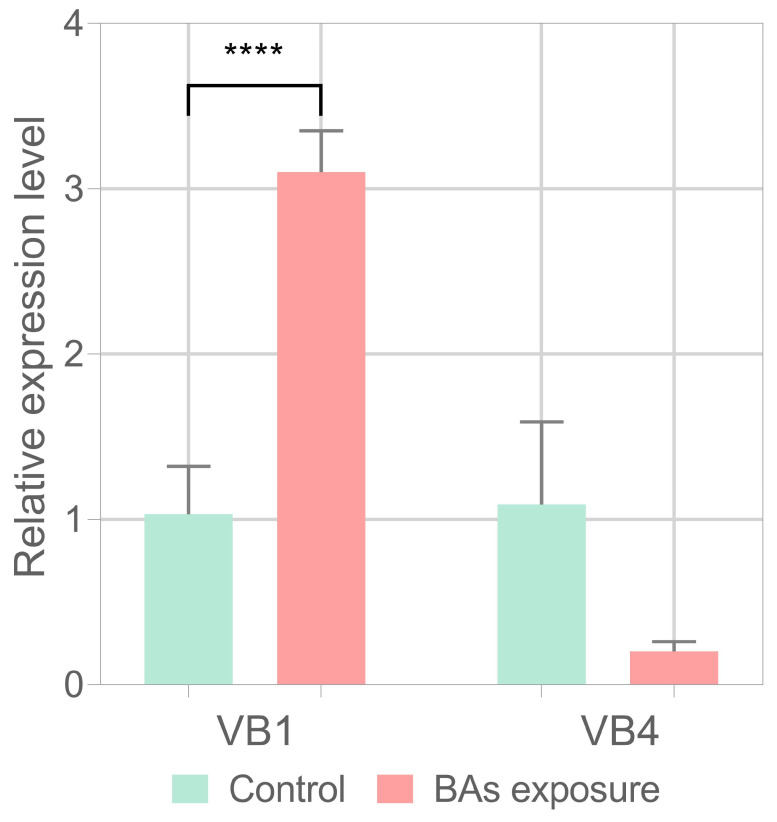
Expression profile of *bsh* genes in *Lacticaseibacillus rhamnosus* VB4 and VB1 in MRS (control condition) and MRS supplemented with 1% (*w*/*v*) BAs mixture. Significant differences were calculated with a Student’s *t*-test and indicated with asterisks, as follow: ****, *p* ≤ 0.0001. The bar graph was plotted with GraphPad Prism v.8.00 software (San Diego, CA, USA).

Furthermore, the VB4 and VB1 strains also strongly differed in the transcriptional regulation of the *bsh* gene. Preliminarily, we assessed the *bsh* gene transcription by end-point PCR and found that both strains actively transcribed the *bsh* gene both in MRS and MRS supplemented with the BAs mixture (Supplementary Figure S5). An RT-qPCR assay was carried out cells in the stationary phase, showing that while the VB4 strain did not significantly change the *bsh* gene expression in the presence of the BAs mixture compared to MRS alone (*p* > 0.05), the VB1 strain significantly increased the *bsh* gene expression when grown on the BAs mixture (*p* < 0.05) (Figure 9).

## 4. Discussion

Since 2009, genomics has contributed to conducting accurate genetic studies of probiotic bacteria, establishing genetic characteristics linked to favorable outcomes as well as those possibly associated with undesirable traits. In combination with in vitro and in vivo assays, it is considered a robust approach for the discovery and characterization of probiotic strains [41,62]. In this study, we presented the genome sequences of *Lcb. rhamnosus* VB4 and VB1 strains isolated from vaginal samples which have showed a remarkable propensity to deconjugate BAs in a preliminary qualitative direct plate assay. Lactobacilli are considered essential in the vaginal environment where they help maintain the vaginal natural acidic pH, inhibit the growth of potentially harmful microbes, and stabilize the microbial balance [63]. In particular, *Lcb. rhamnosus* strains from vaginal microbiota have been extensively investigated for their probiotic adhesion properties and for the capability to synthesize bacteriocins [64]. However, there are no studies related to the investigation of BSH activity in *Lcb. rhamnosus* strains of vaginal origin. 

Safety and genomic stability are essential requirements in probiotics selection. In accordance with regulatory authorities, such as EFSA and FDA, which apply safety guidelines and safety standards on bacterial probiotic strains based on taxonomic classification [60], it is crucial to accurately determine a new strain’s taxonomy before considering its safety and probiotic efficacy. We established unequivocally that VB4 and VB1 strains are *Lcb. rhamnosus* using 16S-rRNA and genome-based phylogenetic analyses. Although many *Lcb. rhamnosus* strains are naturally resistant to vancomycin, this characteristic is an intrinsic phenotype due to the specific structure of cell wall [65]. Congruently, the in silico analysis and genome annotation did not detect any genetic determinants for toxigenic activity and AMR in VB4 and VB1 strains, confirming their safe status. 

Core- and pan-genes have been widely used to investigate bacterial species evolution and to study intra-strain functional differences within species. In accordance with the evidence of this study, the unique genes of VB4 are likely related to its growing environment and metabolic properties, namely carbohydrates metabolism and transport, amino acid transport and metabolism, and cell wall/membrane/envelope biogenesis. Interestingly, both ANIb and clustering analysis of sharing pattern of accessory COG supported that strain VB4 resembles *Lcb. rhamnosus* GG, a probiotic bacterium with BSH activity isolated from human feces, while VB1 genome is related to *Lcb. rhamnosus* GR-1, a vaginal probiotic bacterium. The probiotic strains *Lcb. rhamnosus* GG and GR-1 have been proved to exert serum cholesterol-lowering activity and to be protective against atherosclerotic plaque formation [66,67]; however, these healthy effects have been not related to BSH activity. *Lcb. rhamnosus* GG and GR-1 strains have been recently included in a pan-genome study which identifies eight phylogroups within *Lcb. rhamnosus* [68]. They resulted to belong to phylotypes 1 and 6, respectively, which mainly differed from each other for genes related to adhesion and bacteriocin production. Indeed, these probiotic traits could be investigated in VB4 and VB1 strains in further studies.

Mobile genetic elements, such as plasmids, prophages, gene islands, and insertion elements, play a major role in horizontal gene transfer in bacteria, driving speciation and functional diversification [64]. The identification of prophages belonging to the *Siphoviridae* family (currently listed as morphotypes of the *Caudoviricetes* class [69]), using a Phigaro analysis, revealed a higher number of phage-related genetic elements in the *Lcb. rhamnosus* strain VB4 than VB1. In particular, *Siphoviridae* prophages are double-stranded prophages largely found in the human intestine virome [70], which play a crucial role in bacterial genetic diversity, evolution, and adaptation to changing environments. Previous studies have also documented *Siphoviridae* prophages in *Lcb. rhamnosus* [71,72,73] and in other *Lacticaseibacillus* species [74,75]. Interestingly, the Lc-Nu-like prophage, which has been partially found in the VB1 genome, has also been previously detected in other probiotic strains, including *Lcb. rhamnosus* GG [76]. It has been proposed that LAB strains containing bacteriophages could have positive impacts on human host [77,78]. For instance, the expression of prophage functional genes can confer bacterial cell survival advantages in adverse environments [77]. On the other end, antibiotic resistance genes can be disseminated via phage-mediated transduction. Therefore, it is becoming increasingly evident that prophages should be determined in probiotic genomes for a complete understanding of bacterial physiology, adaptation, and genetic stability.

In addition to prophages, other putative mobile elements, including transposases genes, were found more abundantly in the VB4 genome, compared to the VB1 genome. In addition, the VB4 genome contains a complete type II-A CRISPR–Cas system, which is relatively widespread across the genus *Lactobacillus* [79]. It is expected that CRISPR-positive strains are expected to carry significantly fewer intact prophages than CRISPR-negative strains, as the CRISPR-Cas system acts as anti-phage defense system and inhibits the prophage integration into lactobacilli. However, lactobacilli with a CRISPR-Cas type IIA system, such as *Lcb. rhamnosus* VB4, are more susceptible to temperate phage infections than lactobacilli with a CRISPR-Cas type I/III system [80]. Consistently, the VB4 strain contains a higher number of phage-related genes than the VB1 strain. In contrast, the VB1 strain contains a lower number of putative genetic mobile elements than VB4 and harbors a type I Cas gene. However, the lack of a CRISPR region around the type I Cas gene raises doubts on the authenticity of the CRISPR-Cas structure in the VB1 genome [81].

Additionally, for the genomic characteristics to be fulfilled, potential probiotics must respond to certain phenotypic activities. At the gastrointestinal level, these microorganisms must be able to deconjugate BAs, which are highly toxic due to their cleansing action. The deconjugation of primary BAs catalyzed by bacteria with the BSH^+^ phenotype is considered a pivotal mechanism which assures bacterial fitness and host colonization. Deconjugation products are important precursors of secondary BAs which act as modulators of multiple hosts signaling pathways, mainly involved in body weight maintenance, lipid absorption, and cholesterol metabolism [3]. Consequently, dysregulations of secondary BAs are associated with obesity, hypercholesterolemia, cancer, and *Clostridium difficile* infection [6]. Treatments with BSH-positive probiotics have been shown to increase BSH activity in the gut and confer multiple health benefits to the host, including the reduction in blood cholesterol levels [9]. Therefore, the identification of genes within probiotic genomes involved in BAs deconjugation activity is crucial for discovering new probiotics with BA-modulating properties and potential cholesterol-lowering effects. Even though oral administrations of probiotic lactobacilli have been proven to reduce blood cholesterol levels in animals and humans, the link between bacterial BSH activity and the resulting cholesterol-lowering effect remains poorly understood [3]. Similarly, the data supporting the role of BSH in reducing BAs toxicity and assuring bacterial cells with BAs tolerance are contrasting [13,14,82]. Furthermore, in some probiotic species, such as *Lcb. rhamnosus*, BSH proteins are highly homologous to PVA and are classified under a single family in various public domain databases, including the CBAH family in Pfam, the Ntn-CGH-like family in CDD, and the C59 family in MEROPS [50], leading to possible errors in gene annotation. Recently, the presence of the ‘true’ *bsh* gene has been questioned for some probiotic species, including *Lcb. rhamnosus* [17]. Our results revealed that the VB4 and VB1 strains displayed a distinct BAs deconjugation phenotype without exhibiting any tolerance to PenV. The observed BSH activity is consistent with the presence of the predicted *bsh* gene in the genome, as the only genetic determinants. Remarkably, the BSH protein of strain VB4 was identical in length and amino acid sequence to a *Lcb. rhamnosus* BSH protein which has been experimentally validated by heterologous expression in *E. coli* (AEP69108.1) [23]. We cannot exclude that the predicted BSH proteins of the VB4 and VB1 strains could be active on acyl-homoserine lactones, but, if present, this activity should not contribute to any antibiotic resistance. Similarly, Lambert et al. [20] reported that *bsh2*, *bsh3*, and *bsh4* genes of *Lactiplantibacillus plantarum* WCFS1 encode BSH enzymes active both towards bile salts and two types of acyl-homoserine lactones, without significantly contributing to PenV tolerance. On the other hand, BSH enzyme of *L. gasseri* JCM 5343^T^ was demonstrated to degrade both BAs and PenV [83], suggesting that BSH activity and PenV tolerance should be both evaluated in BSH^+^ probiotic screening.

Although the search for the *bsh* gene is the first step in screening BSH^+^ probiotic candidates, the proposed approach, which integrates genomics and metabolomics, has shown that the high similarity in the sequences of BSH proteins results in neither the same tolerance to BAs nor the same BSH activity in *Lcb. rhamnosus* VB4 and VB1. Indeed, concentrations of BAs slightly higher than the estimated average bile concentration in the human gastrointestinal tract [84] significantly reduced the lag phase of the VB1 strain but compromised its growth rate compared to the VB4 strain, suggesting a greater tolerance of the latter. Furthermore, strain VB1 exhibited a remarkable glyco-specific deconjugation activity, a phenotype expected for BSH^+^ lactobacilli. Like strain VB1, *Lcb. rhamnosus* strain GG is poorly active against TCA and TDCA [25]. The glycine preference of lactobacilli BSH may be due to the higher abundance of glycine-conjugated BAs in human bile and the proposed higher toxicity of these to taurine-conjugated BAs [84,85]. However, Foley and coworkers [12] showed that deconjugated BAs, such as CA, CDCA, and DCA, are more toxic to species *L. acidophilus* and *L. gasseri*, suggesting that conjugated/deconjugated BA–bacterial interaction is more complex than that previously assumed. Similarly, Prete et al. [85] reported that BSH-mediated conversion to more hydrophobic moieties may reduce bacterial growth. 

Unlike VB1, *Lcb. rhamnosus* VB4 strains are also active on tauro-conjugated BAs. The ability to cleave the amide bond between the taurine and steroid moiety has been described in *L. johnsonii* and *L. gasseri* species [10]. Here, it was hypothesized that the BSH activity of the VB4 strain towards taurine-conjugated BAs could have an interesting effect in modulating secondary BAs. Taurine is the limiting factor in bacterial bile acid amidates (BBAAs) synthesis by colonic microbiota [86]. Probiotics with BSH activity on tauro-conjugated BAs could positively affect BBAAs levels. 

BSH proteins of the VB4 and VB1 strains are almost identical, thus the observed difference in BSH activities towards glyco- or tauro-conjugated BA substrates could reflect differences in tolerance to the resulting deconjugated moieties rather than differences in the substrate affinity of the BSH enzymes. Furthermore, the VB4 strain exhibited more BAs-deconjugating activity than VB1 but did not increase the *bsh* gene transcription under BAs exposition, supporting that the strains also differ in *bsh* gene transcriptional regulation. Previous works reported that the exposure of *L. salivarius* and *L. acidophilus* BSH-active strains to bile did not induce the *bsh* gene expression [12,87]. Similarly, Lambert and coworkers [20] reported that the expression of the four *bsh* genes is not induced in the *L. plantarum* WCFS1 strain by exposure to porcine bile, while Bron et al. [88] found that only the *bsh1* gene is strongly upregulated by BAs in this strain. Recently, a transcriptomic study confirmed that all four *bsh* genes are downregulated in *L. plantarum* grown under BAs stress [89]. The lack of correlation between the observed enzymatic activity and the transcriptional regulation of the *bsh* gene in strains VB4 and VB1 could suggest that the BSH+ phenotype involves more gene pathways than the mere BSH activity and that these gene pathways could be different in both tested strains. The presence of conjugated BAs changes the pathways responsible for membrane organization and permeability in *Bifidobacterium longum* [90], while exposure to GCA impacts the expression of genes encoding cell surface proteins and transport proteins in *L. acidophilus* [12]. These results suggest that the ability to eliminate the BAs outside the cell is a key factor in decreasing the detergent effect of deconjugated BAs and assuring a BSH+ phenotype. A time-line transcriptomic analysis coupled with the BA-profiling by metabolomics could help to elucidate this point in the VB4 and VB1 strains in future.

## 5. Conclusions

Probiotics can be one of the promising therapeutic tools for manipulating a host’s BAs profile. The results shown in the present work demonstrated that the VB4 and VB1 strains have very similar BSH proteins but different BSH activity, suggesting that they probably differ in the detoxification system of the resulting unconjugated BAs. Combining bacterial genomics and metagenomic approaches, we demonstrated that strain VB4 is a promising BAs-modulating probiotic candidate and that BSH active phenotype is a complex trait which probably depends on different factors other than the presence of *bsh* gene in *Lcb. rhamnosus* vaginal strains.

## Figures and Tables

**Table 1:** Contigs matching prophage regions and predominant phage sequences predicted in VB4 and VB1 genomes.

Strains	Contigs	Kb	GC%	N° Prophage ORFs	Predominant Phage	Query Coverage (%)	Identity (%)
VB4	2	33.6	46.2	41	*Caudoviricetes* sp. isolate cttX04 (BK021713.1)	83	100
	3	22.3	43.3	10	*Caudoviricetes* sp. isolate ctSOR2 (BK047574.1)	70	100
	12	12.6	45.7	11	*Caudoviricetes* sp. isolate ctRgI1 (BK047375.1)	86	100
VB1	1	23.8	42.7	19	Lc-Nu-like prophage (AY780364.1)	52	99.85
	3	10.2	45.7	10	*Caudoviricetes* sp. isolate ctnST1 (BK026254.1)	97	99.98

## Data Availability

UHPLC/HR-MS data will be made available on request. Raw reads of *Lcb. rhamnosus* VB1 and VB4 genomes have been deposited at the NCBI Sequence Read Archive (SRA) under the accession numbers PRJNA1139681 and PRJNA1139679, respectively.

## Supplementary Materials

The following supporting information can be downloaded at https://www.mdpi.com/article/10.3390/biom15010086/s1, Figure S1: Phylogenetic analysis of 16S rRNA gene sequences of *Lacticaseibacillus* species. The tree was inferred using the Neighbor-Joining method [36] and the Kimura’s two-parameter model [38] with Mega XI software [37]. Strains VB4 and VB1 are shown in bold, while the sequences of reference strains were from the NCBI RefSeq database. A discrete Gamma distribution (shape parameter = 5) was used to model evolutionary rate variation among sites. Bootstrap values are indicated at branch points based on 1000 replications. The trees are drawn in scale, with branch length measured in the number of substitutions per site. Bar represents 0.01 substitutions per nucleotide position. The tree was rooted using the branch leading to four outgroup species *E. faecalis*, *W. coagulans*, *B. subtilis*, and *B. vallismortis*. The tree was visualized with iTOL [39]; Figure S2: KEGG orthology category (KO) distribution of protein-coding genes identified in the genomes of *Lacticaseibacillus rhamnosus* VB4 (A) and VB1 (B), respectively; Figure S3: Organization of the main prophage regions in VB4 (A) and VB1 (B) genomes; Figure S4: Growth curves of VB4 and VB1 strains in presence of 1% (*w*/*v*) BAs mixture (green) compared with control growth conditions (MRS medium; blue). Growth was monitored over the time as mean of OD_600nm_ values in three different biological replicates. Bars (when visible) indicate standard deviation (SD) values of OD_600nm_ measurements. The curves were fitted by non-linear Gompertz model and plotted using v.8.00 software (San Diego, CA, USA); Figure S5: End-point RT-PCR targeting *bsh* gene in BAs-stressed (MRS medium supplemented with 1% (*w*/*v*) BAs) and non-stressed (MRS medium) stationary cells of *Lacticaseibacillus rhamnosus* VB4 (A) and VB1 (B), respectively. Three independent replicates, numbered from R1 to R3, were used. +/− RT indicates addition of reverse transcriptase to the cDNA synthesis reaction. The expected PCR product length was established for each amplicon by using 100 bp DNA Gene Ruler Plus as molecular weight marker. For each RT-PCR reaction gDNA was used as positive control. 16S rRNA gene PCR reactions used as control were omitted. Abbreviations: M, molecular-weight size marker; NTC, negative control; Table S1: Genome assemblies used for ANI matrix calculation; Table S2: List of proteins used as query for BLASTp search of *bsh* genes in VB4 and VB1 genomes [12,20,23,91]; Table S3: Designed primer pairs used in this study; Table S4: General features of *Lacticaseibacillus rhamnosus* VB4 and VB1 genome assemblies; Table S5: Digital DNA-DNA Hybridization (dDDH) values of strains VB4 and VB1 compared with other *Lacticasibacillus* strains; and Table S6: *Cas* genes in VB4 genome. Contigs without *cas* gene were omitted for brevity; Table S7: CRISPR regions in VB4 genome; and Table S8: *Cas* genes in VB1 genome. Contigs without *cas* gene were omitted for brevity; Table S9: CRISPR regions in VB1 genome. Contigs without CRISPR regions were omitted for brevity.

## References

[1] Bustos A.Y., Font de Valdez G., Fadda S., Taranto M.P. (2018). New insights into bacterial bile resistance mechanisms: The role of bile salt hydrolase and its impact on human health. Food Res. Int..

[2] Bourgin M., Kriaa A., Mkaouar H., Mariaule V., Jablaoui A., Maguin E., Rhimi M. (2021). Bile salt hydrolases: At the crossroads of microbiota and human health. Microorganisms.

[3] Agolino G., Pino A., Vaccalluzzo A., Cristofolini M., Solieri L., Caggia C., Randazzo C.L. (2024). Bile salt hydrolase: The complexity behind its mechanism in relation to lowering-cholesterol lactobacilli probiotics. J. Funct. Foods.

[4] Mohanty I., Allaband C., Mannochio-Russo H., El Abiead Y., Hagey L.R., Knight R., Dorrestein P.C. (2024). The changing metabolic landscape of bile acids–keys to metabolism and immune regulation. Nat. Rev. Gastroenterol. Hepatol..

[5] Ridlon J.M., Bajaj J.S. (2015). The human gut sterolbiome: Bile acid-microbiome endocrine aspects and therapeutics. Acta Pharm. Sin. B.

[6] Lee M.H., Nuccio S.P., Mohanty I., Hagey L.R., Dorrestein P.C., Chu H., Raffatellu M. (2024). How bile acids and the microbiota interact to shape host immunity. Nat. Rev. Immunol..

[7] Foley M.H., O’Flaherty S., Barrangou R., Theriot C.M. (2019). Bile salt hydrolases: Gatekeepers of bile acid metabolism and host-microbiome crosstalk in the gastrointestinal tract. PLoS Pathog..

[8] Ridlon J.M., Gaskins H.R. (2024). Another renaissance for bile acid gastrointestinal microbiology. Nat. Rev. Gastroenterol. Hepatol..

[9] Joyce S.A., Shanahan F., Hill C., Gahan C.G. (2014). Bacterial bile salt hydrolase in host metabolism: Potential for influencing gastrointestinal microbe-host crosstalk. Gut Microbes.

[10] Dong Z., Lee B.H. (2018). Bile salt hydrolases: Structure and function, substrate preference, and inhibitor development. Protein Sci..

[11] Yao L., Seaton S.C., Ndousse-Fetter S., Adhikari A.A., DiBenedetto N., Mina A.I., Banks A.S., Bry L., Devlin A.S. (2018). A selective gut bacterial bile salt hydrolase alters host metabolism. eLife.

[12] Foley M.H., O’Flaherty S., Allen G., Rivera A.J., Stewart A.K., Barrangou R., Theriot C.M. (2021). *Lactobacillus* bile salt hydrolase substrate specificity governs bacterial fitness and host colonization. Proc. Natl. Acad. Sci. USA.

[13] Begley M., Hill C., Gahan C.G. (2006). Bile salt hydrolase activity in probiotics. Appl. Environ. Microbiol..

[14] Moser S.A., Savage D.C. (2001). Bile salt hydrolase activity and resistance to toxicity of conjugated bile salts are unrelated properties in lactobacilli. Appl. Environ. Microbiol..

[15] da Silva T.F., Glória R.A., Americo M.F., Freitas A.D.S., de Jesus L.C.L., Barroso F.A.L. (2024). Unlocking the potential of probiotics: A comprehensive review on research, production, and regulation of probiotics. Probiotics Antimicro..

[16] Song Z., Feng S., Zhou X., Song Z., Li J., Li P. (2023). Taxonomic identification of bile salt hydrolase-encoding lactobacilli: Modulation of the enterohepatic bile acid profile. Imeta.

[17] O’Flaherty S., Briner Crawley A., Theriot C.M., Barrangou R. (2018). The *Lactobacillus* Bile salt hydrolase repertoire reveals niche-specific adaptation. mSphere.

[18] McAuliffe O., Cano R.J., Klaenhammer T.R. (2005). Genetic analysis of two bile salt hydrolase activities in *Lactobacillus acidophilus* NCFM. Appl. Environ. Microbiol..

[19] Jayashree S., Pooja S., Pushpanathan M., Rajendhran J., Gunasekaran P. (2014). Identification and characterization of bile salt hydrolase genes from the genome of *Lactobacillus fermentum* MTCC 8711. Appl. Biochem. Biotechnol..

[20] Lambert J.M., Bongers R.S., de Vos W.M., Kleerebezem M. (2008). Functional analysis of four bile salt hydrolase and penicillin acylase family members in *Lactobacillus plantarum* WCFS1. Appl. Environ. Microbiol..

[21] Capurso L. (2019). Thirty years of *Lactobacillus rhamnosus* GG: A review. J. Clin. Gastroenterol..

[22] Rossi F., Amadoro C., Pallotta M.L., Colavita G. (2022). Variability of Genetic Characters Associated with Probiotic Functions in *Lacticaseibacillus* species. Microorganisms.

[23] Kaya Y., Kök M.Ş., Öztürk M. (2017). Molecular cloning, expression and characterization of bile salt hydrolase from *Lactobacillus rhamnosus* E9 strain. Food Biotechnol..

[24] Park S., Kang J., Choi S., Park H., Hwang E., Kang Y.G., Kim A.R., Holzapfel W., Ji Y. (2018). Cholesterol-lowering effect of *Lactobacillus rhamnosus* BFE5264 and its influence on the gut microbiome and propionate level in a murine model. PLoS ONE.

[25] Hernández-Gómez J.G., López-Bonilla A., Trejo-Tapia G., Ávila-Reyes S.V., Jiménez-Aparicio A.R., Hernández-Sánchez H. (2021). In Vitro Bile Salt Hydrolase (BSH) activity screening of different probiotic microorganisms. Foods.

[26] Zafar H., Ain N.U., Alshammari A., Alghamdi S., Raja H., Ali A., Siddique A., Tahir S.D., Akbar S., Arif M. (2022). *Lacticaseibacillus rhamnosus* FM9 and *Limosilactobacillus fermentum* Y57 are as effective as statins at improving blood lipid profile in high cholesterol, high-fat diet model in male wistar rats. Nutrients.

[27] Bolger A.M., Lohse M., Usadel B. (2014). Trimmomatic: A flexible trimmer for Illumina sequence data. Bioinformatics. Bioinformatics.

[28] Wick R.R., Judd L.M., Gorrie C.L., Holt K.E. (2017). Unicycler: Resolving bacterial genome assemblies from short and long sequencing reads. PLoS Comput. Biol..

[29] Seppey M., Manni M., Zdobnov E.M. (2019). BUSCO: Assessing genome assembly and annotation completeness. Meth. Mol. Biol..

[30] Schwengers O., Jelonek L., Dieckmann M.A., Beyvers S., Blom J., Goesmann A. (2021). Bakta: Rapid and standardized annotation of bacterial genomes via alignment-free sequence identification. Microb. Genom..

[31] Grant J.R., Enns E., Marinier E., Mandal A., Herman E.K., Chen C.Y., Graham M., Van Domselaar G., Stothard P. (2023). Proksee: In-depth characterization and visualization of bacterial genomes. Nucleic Acids Res..

[32] Kanehisa M., Sato Y., Morishima K. (2016). BlastKOALA and GhostKOALA: KEGG tools for functional characterization of genome and metagenome sequences. J. Mol. Biol..

[33] Dereeper A., Summo M., Meyer D.F. (2022). PanExplorer: A web-based tool for exploratory analysis and visualization of bacterial pan-genomes. Bioinformatics.

[34] O’Leary N.A., Wright M.W., Brister J.R., Ciufo S., Haddad D., McVeigh R., Rajput B., Robbertse B., Smith-White B., Ako-Adjei D. (2016). Reference sequence (RefSeq) database at NCBI: Current status, taxonomic expansion, and functional annotation. Nucleic Acids Res..

[35] Edgar R.C. (2004). MUSCLE: Multiple sequence alignment with high accuracy and high throughput. Nucleic Acids Res..

[36] Saitou N., Nei M. (1987). The neighbor-joining method: A new method for reconstructing phylogenetic trees. Mol. Biol. Evol..

[37] Tamura K., Stecher G., Kumar S. (2021). MEGA11: Molecular Evolutionary Genetics Analysis Version 11. Mol. Biol. Evol..

[38] Kimura M. (1980). A simple method for estimating evolutionary rates of base substitutions through comparative studies of nucleotide sequences. J. Mol. Evol..

[39] Letunic I., Bork P. (2019). Interactive Tree Of Life (iTOL) v4: Recent updates and new developments. Nucleic Acids Res..

[40] Stackebrandt E. (2006). Taxonomic parameters revisited: Tarnished gold standards. Microb. Today.

[41] Ventura M., O’Flaherty S., Claesson M.J., Turroni F., Klaenhammer T.R., van Sinderen D., O’Toole P.W. (2009). Genome-scale analyses of health-promoting bacteria: Probiogenomics. Nat. Rev. Microbiol..

[42] Starikova E.V., Tikhonova P.O., Prianichnikov N.A., Rands C.M., Zdobnov E.M., Ilina E.N., Govorun V.M. (2020). Phigaro: High-throughput prophage sequence annotation. Bioinformatics.

[43] Malberg Tetzschner A.M., Johnson J.R., Johnston B.D., Lund O., Scheutz F. (2020). In Silico genotyping of *Escherichia coli* isolates for extraintestinal virulence genes by use of whole-genome sequencing data. J. Clin. Microbiol..

[44] Alcock B.P., Huynh W., Chalil R., Smith K.W., Raphenya A.R., Wlodarski M.A., Edalatmand A., Petkau A., A Syed S., Tsang K.K. (2003). CARD 2023: Expanded curation, support for machine learning, and resistome prediction at the comprehensive antibiotic resistance database. Nucleic Acids Res..

[45] Bortolaia V., Kaas R.S., Ruppe E., Roberts M.C., Schwarz S., Cattoir V. (2020). ResFinder 4.0 for predictions of phenotypes from genotypes. J. Antimicrob. Chemother..

[46] Carattoli A., Hasman H. (2020). PlasmidFinder and In Silico pMLST: Identification and typing of plasmid replicons in Whole-Genome Sequencing (WGS). Methods Mol. Biol..

[47] Couvin D., Bernheim A., Toffano-Nioche C., Touchon M., Michalik J., Néron B., Rocha E.P.C., Vergnaud G., Gautheret D., Pourcel C. (2018). CRISPRCasFinder, an update of CRISRFinder, includes a portable version, enhanced performance and integrates search for Cas proteins. Nucleic Acids Res..

[48] Brown C.L., Mullet J., Hindi F., Stoll J.E., Gupta S., Choi M., Keenum I., Vikesland P., Pruden A., Zhang L. (2022). mobileOG-db: A manually curated database of protein families mediating the life cycle of bacterial mobile genetic elements. Appl. Environ. Microbiol..

[49] Papadopoulos J.S., Agarwala R. (2007). COBALT: Constraint-based alignment tool for multiple protein sequences. Bioinformatics.

[50] Daly J.W., Keely S.J., Gahan C.G.M. (2021). Functional and phylogenetic diversity of BSH and PVA Enzymes. Microorganisms.

[51] Waterhouse A.M., Procter J.B., Martin D.M., Clamp M., Barton G.J. (2009). Jalview Version 2—A multiple sequence alignment editor and analysis workbench. Bioinformatics.

[52] Desper R., Gascuel O. (2004). Theoretical foundation of the balanced minimum evolution method of phylogenetic inference and its relationship to weighted least-squares tree fitting. Mol. Biol. Evol..

[53] Grishin N.V. (1995). Estimation of the number of amino acid substitutions per site when the substitution rate varies among sites. J. Mol. Evol..

[54] International Organization of Standardization/International Dairy Federation (2010). (ISO 10932/IDF 223) Milk and Milk Products. Determination of the Minimal Inhibitory Concentration (MIC) of Antibiotics Applicable to Bifidobacteria and Non-Enterococcal Lactic Acid Bacteria (LAB).

[55] Rychen G., Aquilina G., Azimonti G., Bampidis V., de Lourdes Bastos M., Bories G., Chesson A., Cocconcelli P.S., Flachowsky G., EFSA Panel on Additives and Products or Substances used in Animal Feed (FEEDAP) (2018). Guidance on the characterization of microorganisms used as feed additives or as production organisms. EFSA J..

[56] Kahm M., Hasenbrink G., Lichtenberg-Fraté H., Ludwig J., Kschischo M. (2010). grofit: Fitting Biological Growth Curves with R. J. Stat. Softw..

[57] Solieri L., Sola L., Vaccalluzzo A., Randazzo C.L., Martini S., Tagliazucchi D. (2022). Characterization of cell-envelope proteinases from two *Lacticaseibacillus casei* strains isolated from Parmigiano Reggiano cheese. Biology.

[58] Pfaffl M.W., Horgan G.W., Dempfle L. (2002). Relative expression software tool (REST©) for group-wise comparison and statistical analysis of relative expression results in real-time PCR. Nucleic Acids Res..

[59] Karlov D.S., Long S.L., Zeng X., Xu F., Lal K., Cao L., Hayoun K., Lin J., Joyce S.A., Tikhonova I.G. (2023). Characterization of the mechanism of bile salt hydrolase substrate specificity by experimental and computational analyses. Structure.

[60] European Food Safety Authority (EFSA) (2021). EFSA statement on the requirements for whole genome sequence analysis of microorganisms intentionally used in the food chain. EFSA J..

[61] Makarova K.S., Wolf Y.I., Koonin E.V. (2018). Classification and Nomenclature of CRISPR-Cas Systems: Where from Here?. CRISPR J..

[62] Castro-López C., Garcia H.S., Martinez-Avila G.C.G., González-Córdova A.F., Vallejo-Cordoba B., Hernández-Mendoza A. (2021). Genomics-based approaches to identify and predict the health-promoting and safety activities of promising probiotic strains—A probiogenomics review. Trends Food Sci. Technol..

[63] Amabebe E., Anumba D.O. (2018). The vaginal microenvironment: The physiologic role of lactobacilli. Front. Med..

[64] Ceapa C., Davids M., Ritari J., Lambert J., Wels M., Douillard F.P., Smokvina T., de Vos W.M., Knol J., Kleerebezem M. (2016). The variable regions of *Lactobacillus rhamnosus* genomes reveal the dynamic evolution of metabolic and host-adaptation repertoires. Genome Biol. Evol..

[65] Tynkkynen S., Singh K.V., Varmanen P. (1998). Vancomycin resistance factor of *Lactobacillus rhamnosus* GG in relation to enterococcal vancomycin resistance (van) genes. Int. J. Food Microbiol..

[66] Kim B., Park K.Y., Ji Y., Park S., Holzapfel W., Hyun C.K. (2016). Protective effects of *Lactobacillus rhamnosus* GG against dyslipidemia in high-fat diet-induced obese mice. Biochem. Biophys. Res. Commun..

[67] Fang Y., Chen H., Zhang X., Zhang H., Xia J., Ding K., Fang Z. (2019). Probiotic administration of *Lactobacillus rhamnosus* GR-1 attenuates atherosclerotic plaque formation in ApoE-/-mice fed with a high-fat diet. Eur. Rev. Med. Pharmacol. Sci..

[68] Dutra-Silva L., Matteoli F.P., Arisi A.C.M. (2023). Distribution of genes related to probiotic effects across *Lacticaseibacillus rhamnosus* revealed by population structure. Probiotics Antimicrob..

[69] Adriaenssens E.M., Sullivan M.B., Knezevic P., van Zyl L.J., Sarkar B.L., Dutilh B.E., Alfenas-Zerbini P., Łobocka M., Tong Y., Brister J.R. (2020). Taxonomy of prokaryotic viruses: 2018-2019 update from the ICTV bacterial and archaeal viruses subcommittee. Arch. Virol..

[70] Carding S.R., Davis N., Hoyles L. (2017). Review article: The human intestinal virome in health and disease. Aliment. Pharmacol. Ther..

[71] Durmaz E., Miller M.J., Azcarate-Peril M.A., Toon S.P., Klaenhammer T.R. (2008). Genome sequence and characteristics of Lrm1, a prophage from industrial *Lactobacillus rhamnosus* strain M1. Appl. Environ. Microbiol..

[72] Happel A.U., Kullin B.R., Gamieldien H., Jaspan H.B., Varsani A., Martin D., Passmore J.S., Froissart R. (2022). In silico characterisation of putative prophages in *Lactobacillaceae* used in probiotics for vaginal health. Microorganisms.

[73] Wonglapsuwan M., Pahumunto N., Teanpaisan R., Surachat K. (2024). Unlocking the genetic potential of *Lacticaseibacillus rhamnosus* strains: Medical applications of a promising probiotic for human and animal health. Heliyon.

[74] da Silva Barreira D., Lapaquette P., Novion Ducassou J., Couté Y., Guzzo J., Rieu A. (2022). Spontaneous prophage induction contributes to the production of membrane vesicles by the gram-positive bacterium *Lacticaseibacillus casei* BL23. Mbio.

[75] Mohamed H.M., Barzideh Z., Siddiqi M., LaPointe G. (2023). Taxonomy, Sequence Variance and Functional Profiling of the Microbial Community of Long-Ripened Cheddar Cheese Using Shotgun Metagenomics. Microorganisms.

[76] Tuohimaa A., Riipinen K.A., Brandt K., Alatossava T. (2006). The genome of the virulent phage Lc-Nu of probiotic *Lactobacillus rhamnosus*, and comparative genomics with *Lactobacillus casei* phages. Arch. Virol..

[77] Wang X., Kim Y., Ma Q., Hong S.H., Pokusaeva K., Sturino J.M., Wood T.K. (2010). Cryptic prophages help bacteria cope with adverse environments. Nat. Commun..

[78] Nanda A.M., Thormann K., Frunzke J. (2015). Impact of spontaneous prophage induction on the fitness of bacterial populations and host-microbe interactions. J. Bacteriol..

[79] Panahi B., Dehganzad B., Nami Y. (2023). CRISPR-Cas systems feature and targeting phages diversity in *Lacticaseibacillus rhamnosus* strains. Front. Microbiol..

[80] Pei Z., Sadiq F.A., Han X., Zhao J., Zhang H., Ross R.P., Lu W., Chen W. (2021). Comprehensive scanning of prophages in *Lactobacillus*: Distribution, diversity, antibiotic resistance genes, and linkages with CRISPR-Cas systems. Msystems.

[81] Zhang Q., Ye Y. (2017). Not all predicted CRISPR–Cas systems are equal: Isolated cas genes and classes of CRISPR like elements. BMC Bioinform..

[82] Foley M.H., Walker M.E., Stewart A.K., O’Flaherty S., Gentry E.C., Patel S. (2023). Bile salt hydrolases shape the bile acid landscape and restrict *Clostridioides difficile* growth in the murine gut. Nat. Microbiol..

[83] Kusada H., Arita M., Tohno M., Tamaki H. (2022). Bile salt hydrolase degrades β-lactam antibiotics and confers antibiotic resistance on *Lactobacillus paragasseri*. Front. Microbiol..

[84] Corzo G., Gilliland S.E. (1999). Bile salt hydrolase activity of three strains of *Lactobacillus acidophilus*. J. Dairy Sci..

[85] Prete R., Long S.L., Gallardo A.L., Gahan C.G., Corsetti A., Joyce S.A. (2020). Beneficial bile acid metabolism from *Lactobacillus plantarum* of food origin. Sci. Rep..

[86] Rimal B., Collins S.L., Tanes C.E., Rocha E.R., Granda M.A., Solanki S., Hoque N.J., Gentry E.C., Koo I., Reilly E.R. (2024). Bile salt hydrolase catalyses formation of amine-conjugated bile acids. Nature.

[87] Pfeiler E.A., Azcarate-Peril M.A., Klaenhammer T.R. (2007). Characterization of a novel bile-inducible operon encoding a two-component regulatory system in *Lactobacillus acidophilus*. J. Bacteriol..

[88] Bron P.A., Molenaar D., de Vos W.M., Kleerebezem M. (2006). DNA micro-array-based identification of bile-responsive genes in *Lactobacillus plantarum*. J. Appl. Microbiol..

[89] Chen C., Li M., Yu L., Tian F., Zhao J., Chen W., Zhai Q. (2024). Comparative genomics of *Lactiplantibacillus plantarum* reveals the role of the two-component system in response to bile salts stress. Food Biosci..

[90] Sánchez B., Champomier-Vergès M.C., Anglade P., Baraige F., de Los Reyes-Gavilán C.G., Margolles A., Zagorec M. (2005). Proteomic analysis of global changes in protein expression during bile salt exposure of *Bifidobacterium longum* NCIMB 8809. J. Bacteriol..

[91] Zhang W.Y., Wu R.N., Sun Z.H., Meng H., Zhang H.P. (2009). Molecular cloning and characterization of bile salt hydrolase in *Lactobacillus casei* Zhang. Ann. Microbiol..

