# Supplementary material for: Genome Mining and Characterization of Two Novel Lacticaseibacillus rhamnosus Probiotic Candidates with Bile Salt Hydrolase Activity
Source: Biomolecules. 2025 Jan 8;15(1):86. doi: 10.3390/biom15010086 (PMC11763831; doi:10.3390/biom15010086)
Supplement: Supplementary file 1 [file biomolecules-15-00086-s001.zip › Supplementary Figures_Biomolecules_proof.pdf]

Supplementary Figures

Genome mining and characterization of two novel *Lacticaseibacillus rhamnosus* probiotic candidates with bile salt hydrolase activity

Gianluigi Agolino [1], Marianna Cristofolini [2], Amanda Vaccalluzzo [1], Davide Tagliazucchi [2], Alice Cattivelli [2], Alessandra Pino [1,3]*, Cinzia Caggia [1,3], Lisa Solieri [2,*] and Cinzia Lucia Randazzo[1,3]

[1] Department of Agriculture, Food and Environment, University of Catania, 95123 Catania, Italy; gianluigi.agolino@phd.unict.it (G.A.); amanda.vaccalluzzo@unict.it (A.V.); alessandra.pino@unict.it (A.P.); ccaggia@unict.it (C.G.); cinzia.randazzo@unict.it (C.L.R.)

[2] Department of Life Science, University of Modena and Reggio Emilia, 42122 Reggio Emilia, Italy; marianna.cristofolini@unimore.it (M.C.); davide.tagliazucchi@unimore.it (D.T.); alice.cattivelli@unimore.it (A. C.); lisa.solieri@unimore.it (L.S.)

[3] ProBioEtna srl, Spin Off of the University of Catania, Santa Sofia Street, 100, 95123 Catania, Italy; alessandra.pino@unict.it (A.P.); ccaggia@unict.it (C.G.); cinzia.randazzo@unict.it (C.L.R.)

* Correspondence: alessandra.pino@unict.it (A.P.); lisa.solieri@unimore.it (L.S.)

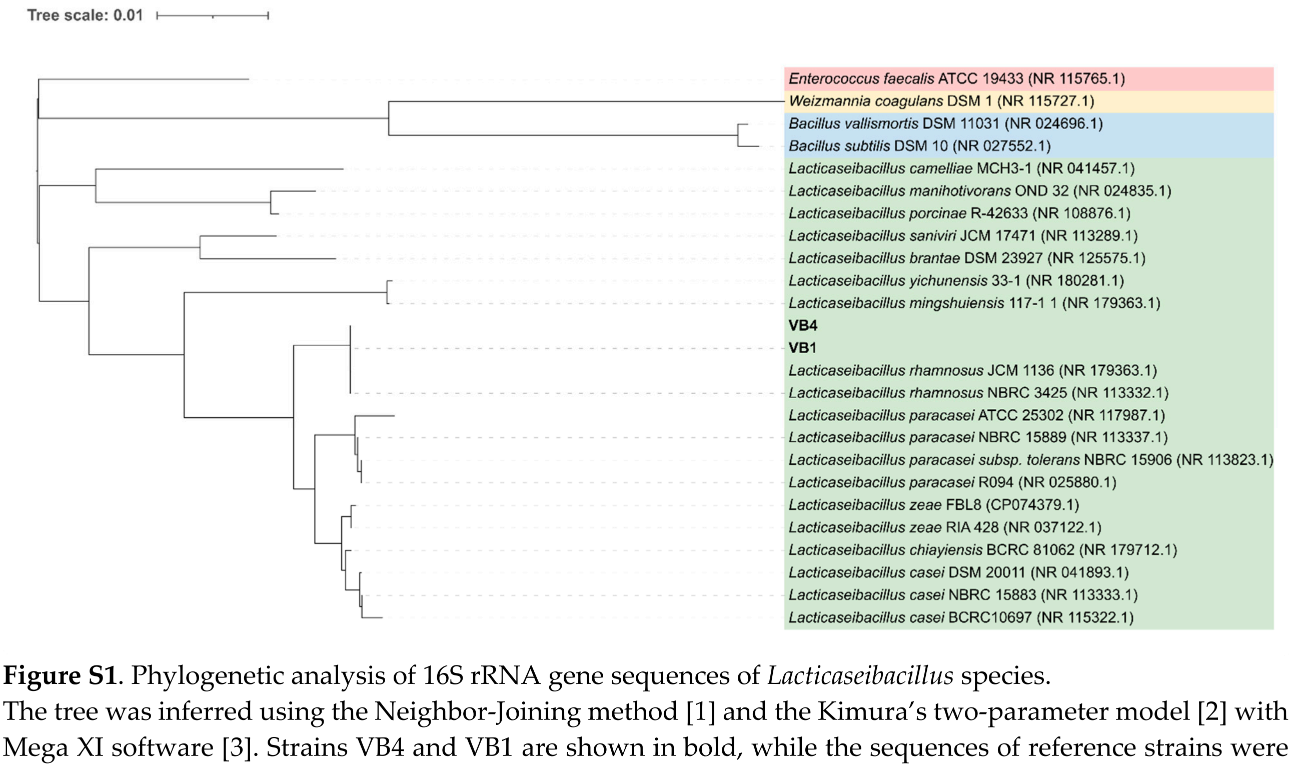

Figure S1. Phylogenetic analysis of 16S rRNA gene sequences of *Lacticaseibacillus* species.
The tree was inferred using the Neighbor-Joining method [1] and the Kimura's two-parameter model [2] with Mega XI software [3]. Strains VB4 and VB1 are shown in bold, while the sequences of reference strains were from the NCBI RefSeq database. A discrete Gamma distribution (shape parameter = 5) was used to model evolutionary rate variation among sites. Bootstrap values are indicated at branch points based on 1,000 replications. The trees are drawn in scale, with branch length measured in the number of substitutions per site. Bar represents 0.01 substitutions per nucleotide position. The tree was rooted using the branch leading to four outgroup species *E. faecalis*, *W. coagulans*, *B. subtilis*, and *B. vallismortis*. The tree was visualized with iTOL version 7 [4].

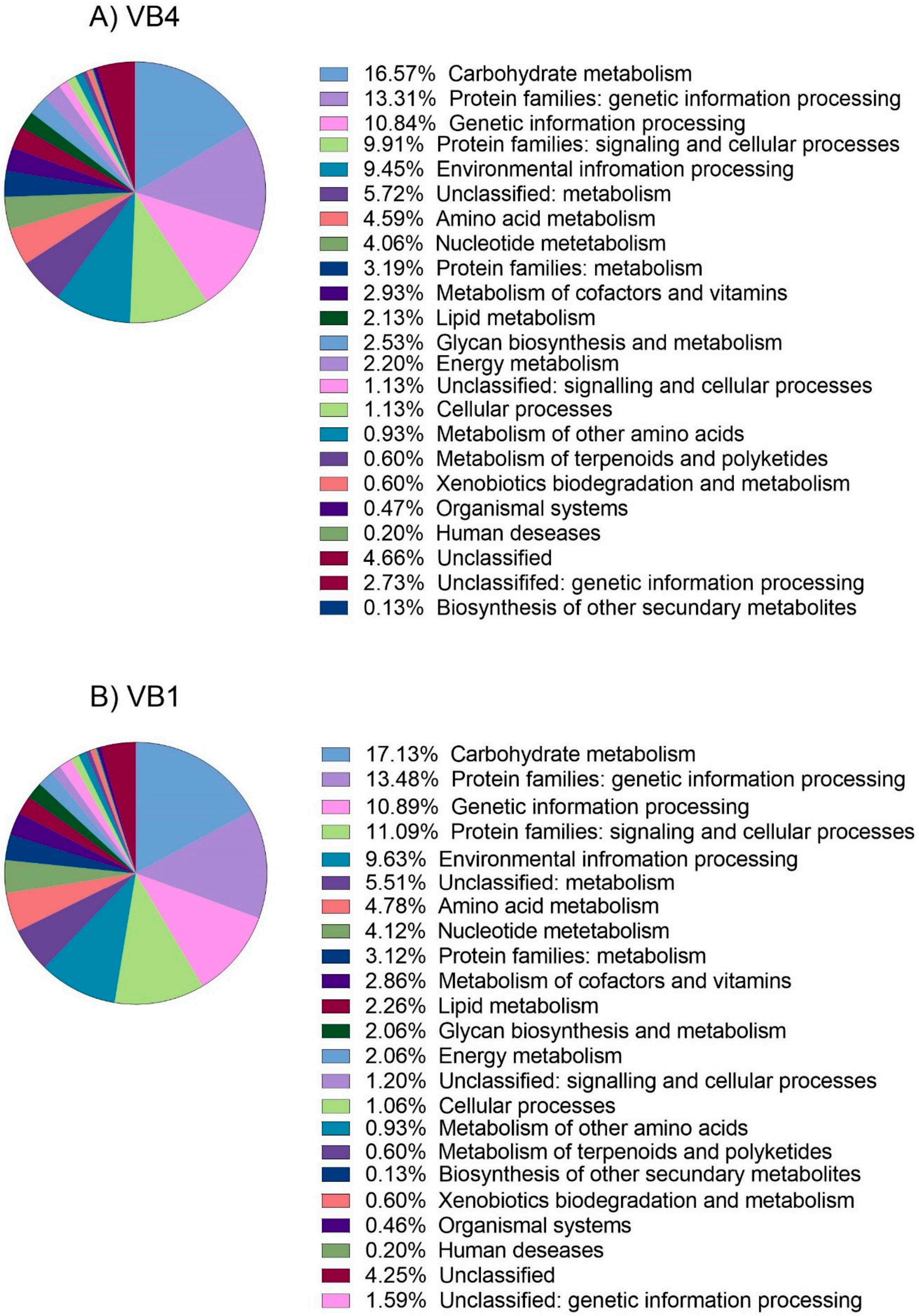

Figure S2. Distribution of KEGG orthology (KO) categories of identified protein-coding genes in the *Lacticaseibacillus rhamnosus* VB4 (**A**) and VB1 (**B**) genomes, respectively.

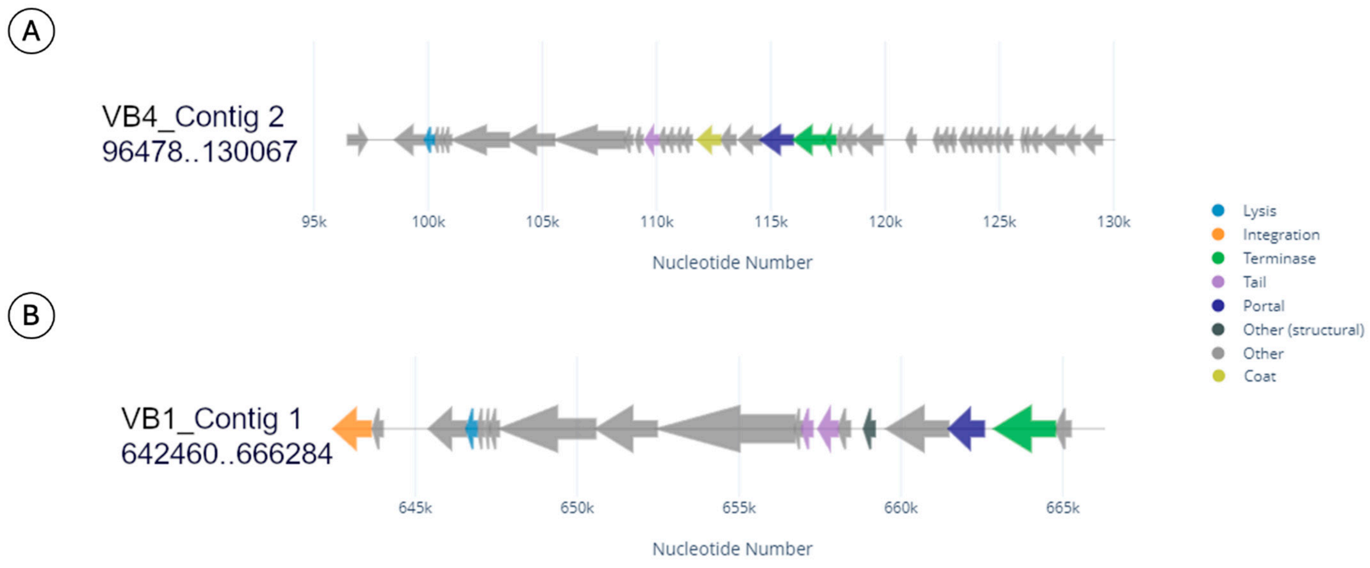

Figure S3. Organization of the main prophage regions in VB4 (**A**) and VB1 (**B**) genomes.

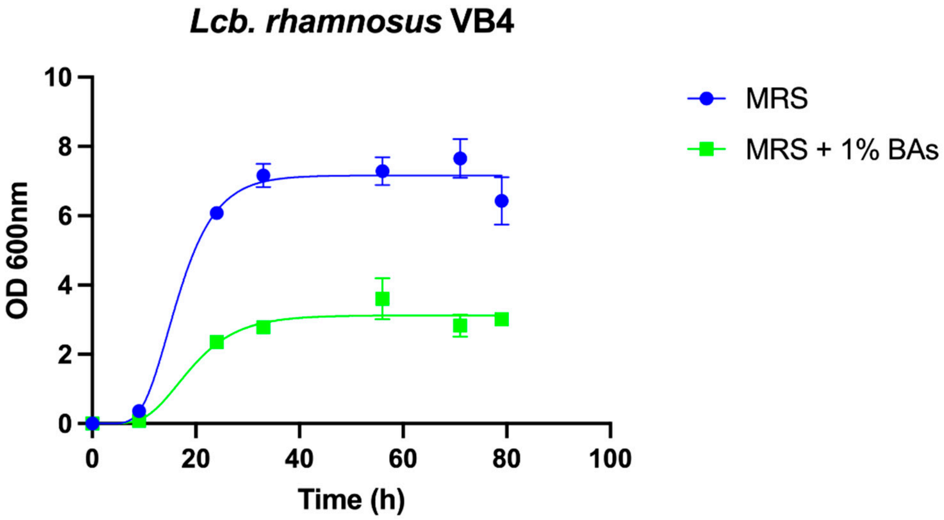

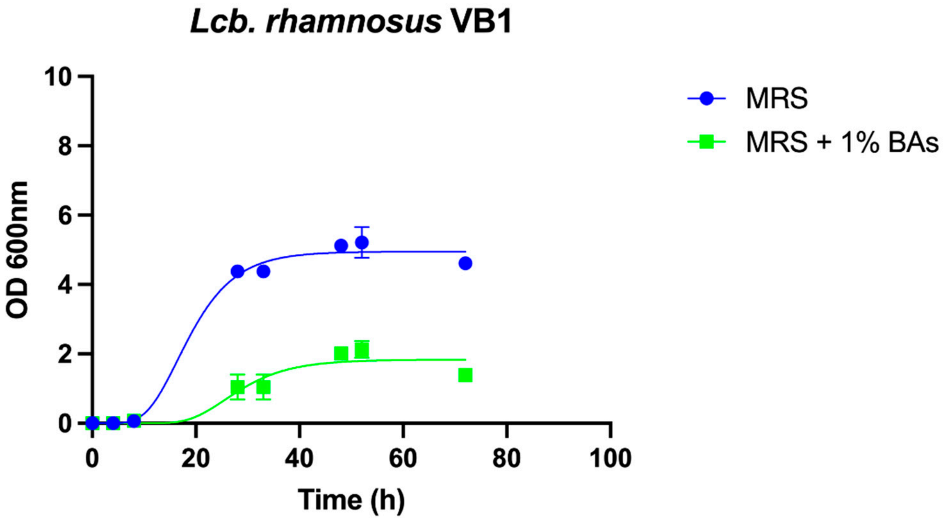

Figure S4. Growth curves of VB4 and VB1 strains in presence of 1% (w/v) BAs mixture (green) compared with control growth conditions (MRS medium; blue). Growth was monitored over the time as mean of OD_{600nm} values in three different biological replicates. Bars (when visible) indicate standard deviation (SD) values of OD_{600nm} measurements. The curves were fitted by non-linear Gompertz model and plotted using GraphPad v.8.00 software (San Diego, CA, USA).

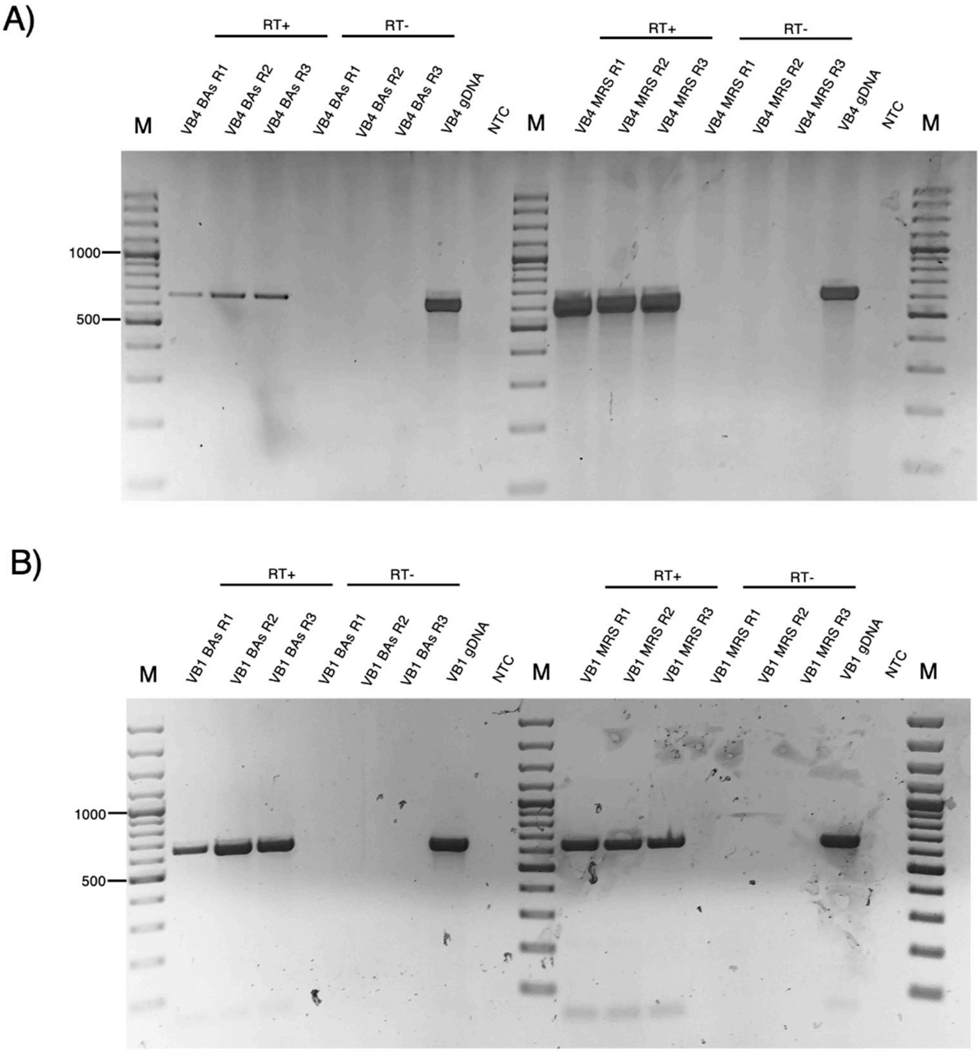

Figure S5. End-point RT-PCR targeting *bsh* gene in BAs-stressed (MRS medium supplemented with 1% (w/v) BAs) and non-stressed (MRS medium) stationary cells of *Lacticaseibacillus rhamnosus* VB4 (**A**) and VB1 (**B**), respectively. Three independent replicates, numbered from R1 to R3, were used. +/- RT indicates addition of reverse transcriptase to the cDNA synthesis reaction. The expected PCR product length was established for each amplicon by using 100 bp DNA Gene Ruler Plus as molecular weight marker. For each RT-PCR reaction gDNA was used as positive control. 16S rRNA gene PCR reactions used as control were omitted. Abbreviations: M, molecular-weight size marker; NTC, negative control.
