# Supplementary material for: Genome Mining and Characterization of Two Novel Lacticaseibacillus rhamnosus Probiotic Candidates with Bile Salt Hydrolase Activity
Source: Biomolecules. 2025 Jan 8;15(1):86. doi: 10.3390/biom15010086 (PMC11763831; doi:10.3390/biom15010086)
Supplement: Supplementary file 1 [file biomolecules-15-00086-s001.zip › Supplementary Tables_Biomolecules_proof.pdf]

Supplementary Tables

Genome mining and characterization of two novel *Lacticaseibacillus rhamnosus* probiotic candidates with bile salt hydrolase activity

Gianluigi Agolino [1], Marianna Cristofolini [2], Amanda Vaccalluzzo [1], Davide Tagliazucchi [2], Alice Cattivelli [2], Alessandra Pino [1,3*], Cinzia Caggia [1,3], Lisa Solieri [2,*] and Cinzia Lucia Randazzo[1,3]

[1] Department of Agriculture, Food and Environment, University of Catania, 95123 Catania, Italy; gianluigi.agolino@phd.unict.it (G.A.); amanda.vaccalluzzo@unict.it (A.V.); alessandra.pino@unict.it (A.P.); ccaggia@unict.it (C.G.); cinzia.randazzo@unict.it (C.L.R.)

[2] Department of Life Science, University of Modena and Reggio Emilia, 42122 Reggio Emilia, Italy; marianna.cristofolini@unimore.it (M.C.); davide.tagliazucchi@unimore.it (D.T.); alice.cattivelli@unimore.it (A. C.); lisa.solieri@unimore.it (L.S.)

[3] ProBioEtna srl, Spin Off of the University of Catania, Santa Sofia Street, 100, 95123 Catania, Italy; alessandra.pino@unict.it (A.P.); ccaggia@unict.it (C.G.); cinzia.randazzo@unict.it (C.L.R.)

* Correspondence: alessandra.pino@unict.it (A.P.); lisa.solieri@unimore.it (L.S.)

Table S1. Genome assemblies used for ANI matrix calculation.

Specie	Strain	GenBank accession number
Lacticaseibacillus casei	N87	GCA_001013375.1
Lacticaseibacillus casei	DSM 20011 = JCM 1134 = ATCC 393 JCM 1134 [T]	GCA_000829055.1
Lacticaseibacillus casei	DSM 20011 = JCM 1134 = ATCC 393 [T]	GCA_000615205.1
Lacticaseibacillus casei	12A	GCA_000309565.2
Lacticaseibacillus casei	21/1	GCA_000309585.1
Lacticaseibacillus casei	A2-362	GCA_000510825.1
Lacticaseibacillus casei	UW4	GCA_000309745.1
Lacticaseibacillus huelsenbergensis	DSM 115425 [T]	GCA_029726355.1
Lacticaseibacillus mingshuiensis	117-1 [T]	GCA_016861785.1
Lacticaseibacillus paracasei	BL23	GCA_964065205.1
Lacticaseibacillus paracasei	ATCC 334	GCA_000014525.1
Lacticaseibacillus paracasei	L9	GCA_964065205.1
Lacticaseibacillus paracasei	CAUH35	GCA_001191565.1
Lacticaseibacillus paracasei	Zhang	GCA_000019245.3
Lacticaseibacillus paracasei	LOCK919	GCA_000418515.1
Lacticaseibacillus paracasei	LC2W	GCA_000194785.1
Lacticaseibacillus paracasei subsp. *paracasei*	ATCC 25302 = DSM 5622 = JCM 8130 ATCC 25302 [T]	GCA_004354655.1
*Lacticaseibacillus paracasei s*ubsp. *paracasei*	JCM 8130 [T]	GCA_000159495.1
Lacticaseibacillus rhamnosus	ATCC 8530	GCA_000233755.1
Lacticaseibacillus rhamnosus	DSM 20021 = JCM 1136 = NBRC 3425 DSM 20021 [T]	GCA_007990855.1
Lacticaseibacillus rhamnosus	GG ATCC 53103	GCA_000011045.1
Lacticaseibacillus rhamnosus	Lc 705	GCA_000026525.1
Lacticaseibacillus rhamnosus	LOCK900	GCA_000418475.1
Lacticaseibacillus rhamnosus	LOCK908	GCA_000418495.1
Lacticaseibacillus rhamnosus	BPL15	GCA_001368735.1
Lacticaseibacillus rhamnosus	LMS2-1	GCA_000160175.1

Table S2. List of proteins used as query for BLASTp search of *bsh* genes in VB4 and VB1 genomes.

Accession number	Locus tag	Old locus tag	Coordinates	Encoded Protein	Source Organism	Reference
NC_004567.2	LP_RS14790	lp_0067	3.154.512..3.155.486	Bile salt hydrolase 1 (WP_011102211.1)	*Lactiplantibacillus plantarum* WCFS1	[20]
NC_004567.2	LP_RS00270	lp_0067	64.823..65.839	Bile salt hydrolase 2 (WP_011100864.1)		
NC_004567.2	LP_RS10935	lp_2572	2.290.117..2.291.070	Bile salt hydrolase 3 (WP_011101831.1)		
NC_004567.2	PL_RS14050	lp_3362	2.987.554..2.988.540	Bile salt hydrolase 4 (WP_003643511.1)		
CP156988.1	ABG771_04540	-	869290..870252	Bile salt hydrolase A (XBE42563.1)	*Lactobacillus acidophilus* NCK1909	[91]
CP156988.1	ABG771_05415	-	1058236..1059213	bile salt hydrolase B (XBE40978.1)		
NC_008530.1	LGAS_RS00260	LGAS_0054	70380..69430	Bile salt hydrolase A (7SVF)	*Lactobacillus gasseri* ATCC 33323 (JCM 1131)	
NC_008530.1	LGAS_RS04710	LGAS_0959	960746..961723	Bile salt hydrolase B (7SVH)		
ASM1924v3	LCAZH_RS02445	LCAZH_0447	448786..449802	ACC93573.1 (WP_003583296.1)	*Lacticaseibacillus paracasei* Zhang	[92]
ANQ47241.1	-	-	-	-	*Lacticaseibacillus rhamnosus* E9	[23]

Table S3. Primers used in this study.

PCR assay	Target gene	Primer	Sequence (5'->3')	Lenght (bp)	T_m	PCR product lenght (bp)
end-point PCR	*bsh*	Bsh_rha_F3	ACGGCTACTTGGAAGACTAAAT	22	62	643
		Bsh_rha_R3	CATTGTAAAGCACGCGGATAC	21	62	
RT-qPCR	*bsh*	Bsh_rha_qF2	GGAATACGGGTGGCATACAA	20	63	136
		Bsh_rha_qR2	CAGGCCAAACATGCCATAAC	20	63	
	16S	16S_F1	GTAGGTGGCAAGCGTTATCC	20	62.8	101
		16S_R1	GATGCGCTTCCTCGGTTAAG	20	63	

Table S4. General features of *Lacticaseibacillus rhamnosus* VB4 and VB1 genome assemblies.

Features	VB4	VB1
Size (bp)	2,926,936	2,917,389
Contigs	70	19
GC (%)	46.63	46.65
N50	111,122	406,064
Largest contig (bp)	222,067	1,025,915
CDS	2769	2704
Gene average length (bp)	903	921
tRNA	45	46
rRNA	4	2
tmRNA (ssrA)	1	1
ncRNA	4	7
oriC	1	2

Table S5. Digital DNA-DNA Hybridization (dDDH) values of strains VB4 and VB1 compared with other *Lacticasibacillus* strains.

Query strain	Subject strain	dDDH (d0, in %)	C.I. (d0, in %)	dDDH (d4, in %)	C.I. (d4, in %)	dDDH (d6, in %)	C.I. (d6, in %)	G+C content difference (in %)
VB4	*Lacticaseibacillus rhamnosus* JCM 1136	79,9	[75.9 - 83.3]	76,7	[73.7 - 79.5]	82,2	[78.9 - 85.1]	0,05
VB4	*Lacticaseibacillus casei* ATCC 393	25,2	[21.8 - 28.8]	24,1	[21.8 - 26.6]	23,9	[21.0 - 27.0]	1,25
VB4	*Lacticaseibacillus huelsenbergensis* DSM 115425T	25,4	[22.0 - 29.0]	23,6	[21.3 - 26.1]	23,9	[21.1 - 27.0]	1,3
VB4	*Lacticaseibacillus paracasei* JCM 8130	20,2	[17.0 - 23.8]	23,5	[21.2 - 25.9]	19,7	[17.0 - 22.8]	0,08
VB4	*Lacticaseibacillus zeae* KCTC 3804	25,5	[22.2 - 29.2]	23,3	[21.0 - 25.8]	24	[21.2 - 27.1]	1,12
VB4	*Lacticaseibacillus chiayiensis* BCRC 81062	25,5	[22.1 - 29.1]	22,9	[20.6 - 25.3]	23,9	[21.0 - 27.0]	0,51
VB4	*Lacticaseibacillus casei* DSM 20011	20	[16.8 - 23.6]	22,6	[20.3 - 25.0]	19,5	[16.8 - 22.5]	0,19
VB4	*Lacticaseibacillus paracasei subsp. tolerans* DSM 20258	19,7	[16.5 - 23.3]	22,5	[20.3 - 25.0]	19,2	[16.5 - 22.2]	0,21
VB4	*Lacticaseibacillus paracasei* ATCC 25302	19,8	[16.7 - 23.4]	22,4	[20.1 - 24.8]	19,3	[16.6 - 22.4]	0,12
VB1	*Lacticaseibacillus rhamnosus* JCM 1136	97,8	[96.3 - 98.6]	98,7	[98.0 - 99.1]	98,8	[98.0 - 99.3]	0,03
VB1	*Lacticaseibacillus casei* ATCC 393	25,2	[21.9 - 28.8]	23,9	[21.6 - 26.3]	23,8	[21.0 - 26.9]	1,23
VB1	*Lacticaseibacillus huelsenbergensis* DSM 115425T	25,6	[22.3 - 29.3]	23,3	[21.0 - 25.8]	24,1	[21.2 - 27.2]	1,29
VB1	*Lacticaseibacillus zeae* KCTC 3804	25,1	[21.7 - 28.7]	23,3	[21.0 - 25.7]	23,6	[20.8 - 26.7]	1,1
VB1	*Lacticaseibacillus chiayiensis* BCRC 81062	24,9	[21.6 - 28.6]	22,9	[20.6 - 25.4]	23,5	[20.6 - 26.6]	0,49
VB1	*Lacticaseibacillus paracasei* JCM 8130	19,6	[16.4 - 23.2]	22,2	[19.9 - 24.6]	19,1	[16.4 - 22.1]	0,1
VB1	*Lacticaseibacillus paracasei subsp. tolerans* DSM 20258	19,6	[16.4 - 23.1]	22,1	[19.8 - 24.5]	19,1	[16.4 - 22.1]	0,23
VB1	*Lacticaseibacillus paracasei* ATCC 25302	19,7	[16.5 - 23.3]	22	[19.7 - 24.4]	19,2	[16.5 - 22.2]	0,14
VB1	*Lacticaseibacillus casei* DSM 20011	19,8	[16.6 - 23.4]	21,8	[19.5 - 24.2]	19,3	[16.5 - 22.3]	0,21
VB1	*Lacticaseibacillus mingshuiensis* JCM 33567	12,9	[10.2 - 16.2]	20,4	[18.1 - 22.8]	13,3	[10.9 - 16.0]	8,52

Table S6. *Cas* genes in VB4 genome. Contigs without *cas* gene were omitted for brevity.

#SequenceID	Cas-type/subtype	Gene status	System	Type	Begin	End	Strand	Other information
contig_9_29	csn2_TypeIIA	mandatory	CAS-TypeIIA	CDS	29106	29786	-	ID=1_29;partial=00;start_type=TTG;rbs_motif=AGGA;rbs_spacer=5-10bp;gc_cont=0.402;conf=100.00;score=78.52;cscore=74.54;sscore=3.97;rscore=10.77;uscore=0.11;tscore=-8.20;
contig_9_30	cas2_TypeI-II-III	accessory	CAS	CDS	29783	30088	-	ID=1_30;partial=00;start_type=ATG;rbs_motif=GGAG/GAGG;rbs_spacer=5-10bp;gc_cont=0.438;conf=99.99;score=42.04;cscore=23.07;sscore=18.98;rscore=9.74;uscore=5.52;tscore=3.72;
contig_9_31	cas1_TypeII	accessory	CAS-TypeIIU	CDS	30066	30971	-	ID=1_31;partial=00;start_type=ATG;rbs_motif=GGA/GAG/AGG;rbs_spacer=5-10bp;gc_cont=0.447;conf=100.00;score=92.61;cscore=87.24;sscore=5.37;rscore=0.78;uscore=1.52;tscore=3.72;
contig_9_32	cas9_TypeII	mandatory	CAS-TypeIIU	CDS	31177	35268	-	ID=1_32;partial=00;start_type=ATG;rbs_motif=GGAGG;rbs_spacer=5-10bp;gc_cont=0.423;conf=99.99;score=524.92;cscore=502.47;sscore=22.45;rscore=16.30;uscore=3.08;tscore=3.72;

Table S7. CRISPR regions in VB4 genome. Contigs without CRISPR regions were omitted for brevity.

VB4		
**Sequence**	contig_9	contig_10
**Sequence_basename**	contig_9	contig_10
**Duplicated_Spacers**	0	0
**CRISPR_Id**	contig_9_1	contig_10_1
**CRISPR_Start**	27532	59245
**CRISPR_End**	29085	59346
**CRISPR_Length**	1553	101
**Potential_Orientation (AT%)**	Reverse	Unknown
**CRISPRDirection**	Unknown	Unknown
**Consensus_Repeat**	GCTCTTGAACTGATTGATCT GACATCTACCTGAGAC	GGGTAAAACCCTTCCAAT CTCCAACTTCATTATACC
**Repeat_ID (CRISPRdb)**	Unknown	Unknown
**Nb_CRISPRs_with_same_Repeat (CRISPRdb)**	0	0
**Repeat_Length**	36	36
**Spacers_Nb**	23	1
**Mean_size_Spacers**	30	30
**Standard_Deviation_Spacers**	0	0
**Nb_Repeats_matching_Consensus**	21	2
**Ratio_Repeats_match/TotalRepeat**	0.875	1
**Conservation_Repeats (% identity)**	91.6666666666667	100
**EBcons_Repeats**	97.2307923074907	100
**Conservation_Spacers (% identity)**	0	100
**EBcons_Spacers**	0	100
**Repeat_Length_plus_mean_size_Spacers**	66	66
**Ratio_Repeat/mean_Spacers_Length**	1.2	1.2
**CRISPR_found_in_DB (if sequence IDs are similar)**	0	0
**Evidence_Level**	4	1

Table S8. *Cas* genes in VB1 genome. Contigs without *cas* gene were omitted for brevity.

#SequenceID	Cas-type/subtype	Gene status	System	Type	Begin	End	Strand	Other_information
contig_9_38	cas3_TypeI	forbidden	CAS	CDS	41617	42963	+	ID=1_38;partial=00;start_type=ATG;rbs_motif=AGxAGG/AGGxGG;rbs_spacer=3-4bp;gc_cont=0.491;conf=100.00;score=147.15;cscore=131.40;sscore=15.75;rscore=7.31;uscore=3.24;tscore=3.99;

Table S9. CRISPR regions in VB1 genome. Contigs without CRISPR regions were omitted for brevity.

**VB1**	
**Sequence**	contig_1
**Sequence_basename**	contig_1
**Duplicated_Spacers**	0
**CRISPR_Id**	contig_1_1
**CRISPR_Start**	88741
**CRISPR_End**	88886
**CRISPR_Length**	145
**Potential_Orientation (AT%)**	Unknown
**CRISPRDirection**	Unknown
**Consensus_Repeat**	GCGTGATGGCCGGTCTTTGGCCATTGCGCCCAAGGTCCTTACA
**Repeat_ID (CRISPRdb)**	Unknown
**Nb_CRISPRs_with_same_Repeat (CRISPRdb)**	0
**Repeat_Length**	43
**Spacers_Nb**	1
**Mean_size_Spacers**	60
**Standard_Deviation_Spacers**	0
**Nb_Repeats_matching_Consensus**	1
**Ratio_Repeats_match/TotalRepeat**	0.5
**Conservation_Repeats (% identity)**	97.6744186046512
**EBcons_Repeats**	97.6744186046512
**Conservation_Spacers (% identity)**	100
**EBcons_Spacers**	100
**Repeat_Length_plus_mean_size_Spacers**	103
**Ratio_Repeat/mean_Spacers_Length**	0.716666666666667
**CRISPR_found_in_DB (if sequence IDs are similar)**	0
**Evidence_Level**	1
