# Supplementary figures and images for: Genome Mining and Characterization of Two Novel Lacticaseibacillus rhamnosus Probiotic Candidates with Bile Salt Hydrolase Activity
Source: Biomolecules. 2025 Jan 8;15(1):86. doi: 10.3390/biom15010086 (PMC11763831; doi:10.3390/biom15010086)

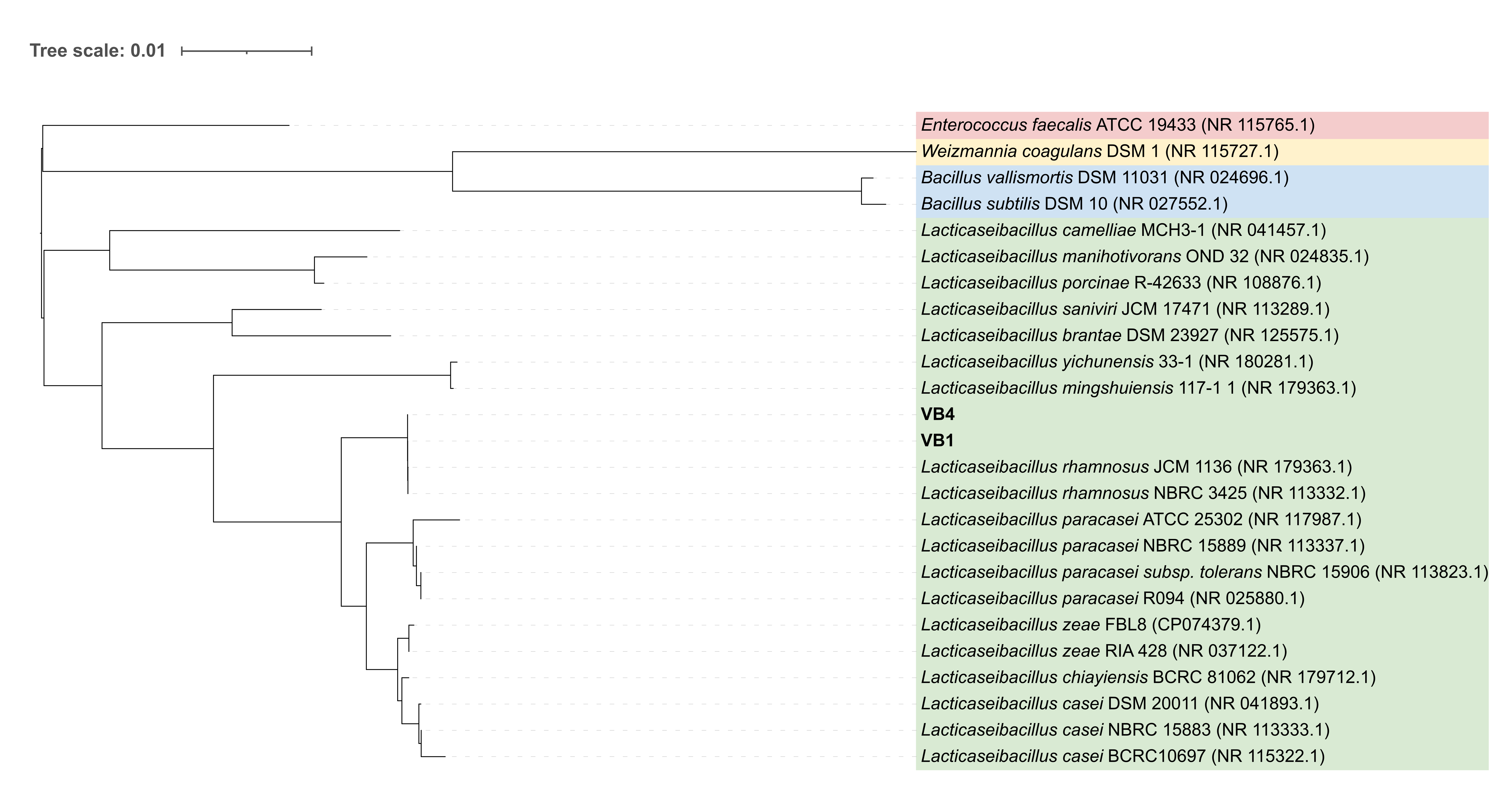

Supplement: Supplementary file 1 [file biomolecules-15-00086-s001.zip › Figure S1.tiff]

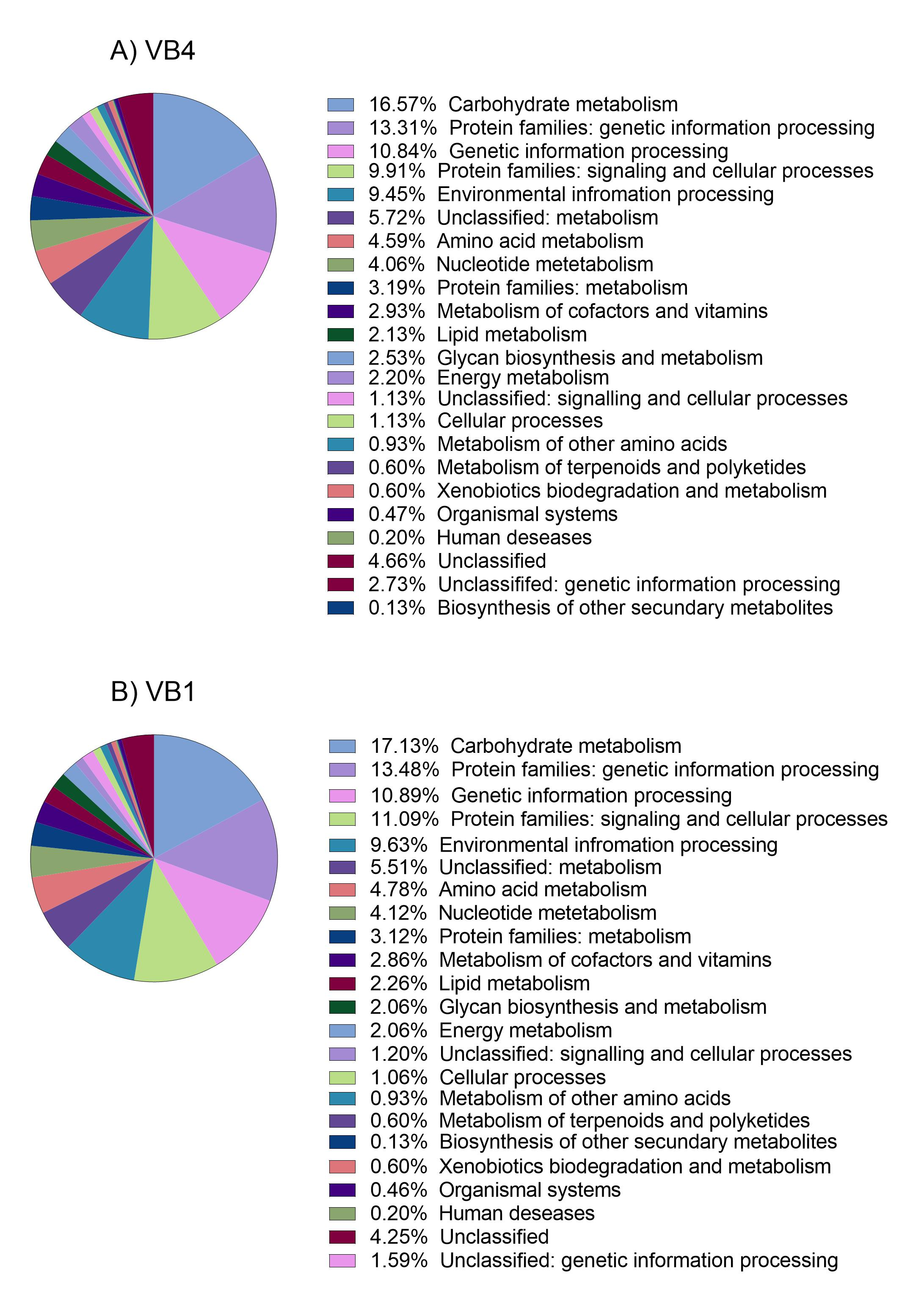

Supplement: Supplementary file 1 [file biomolecules-15-00086-s001.zip › Figure S2.tiff]

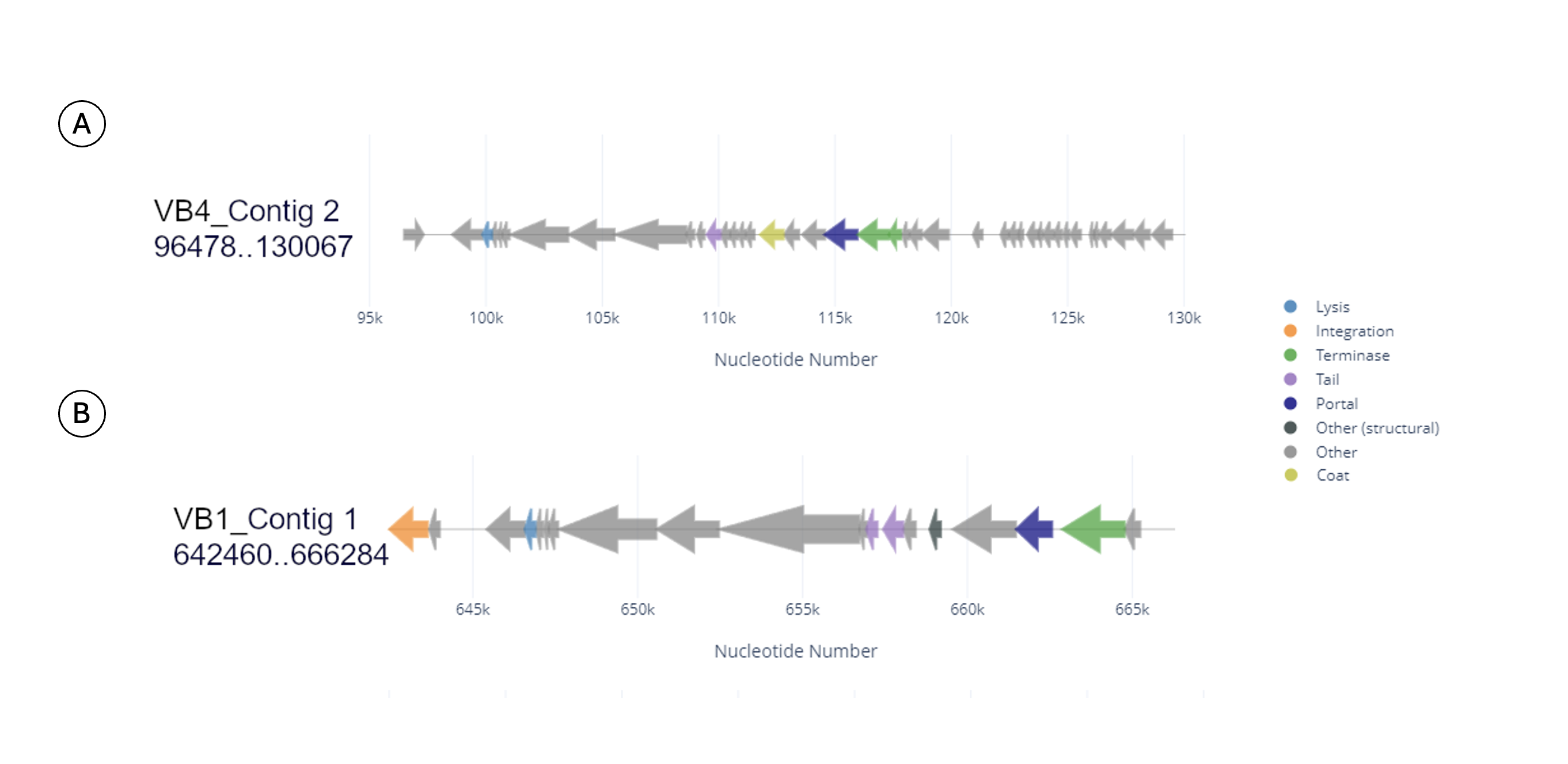

Supplement: Supplementary file 1 [file biomolecules-15-00086-s001.zip › Figure S3.tiff]

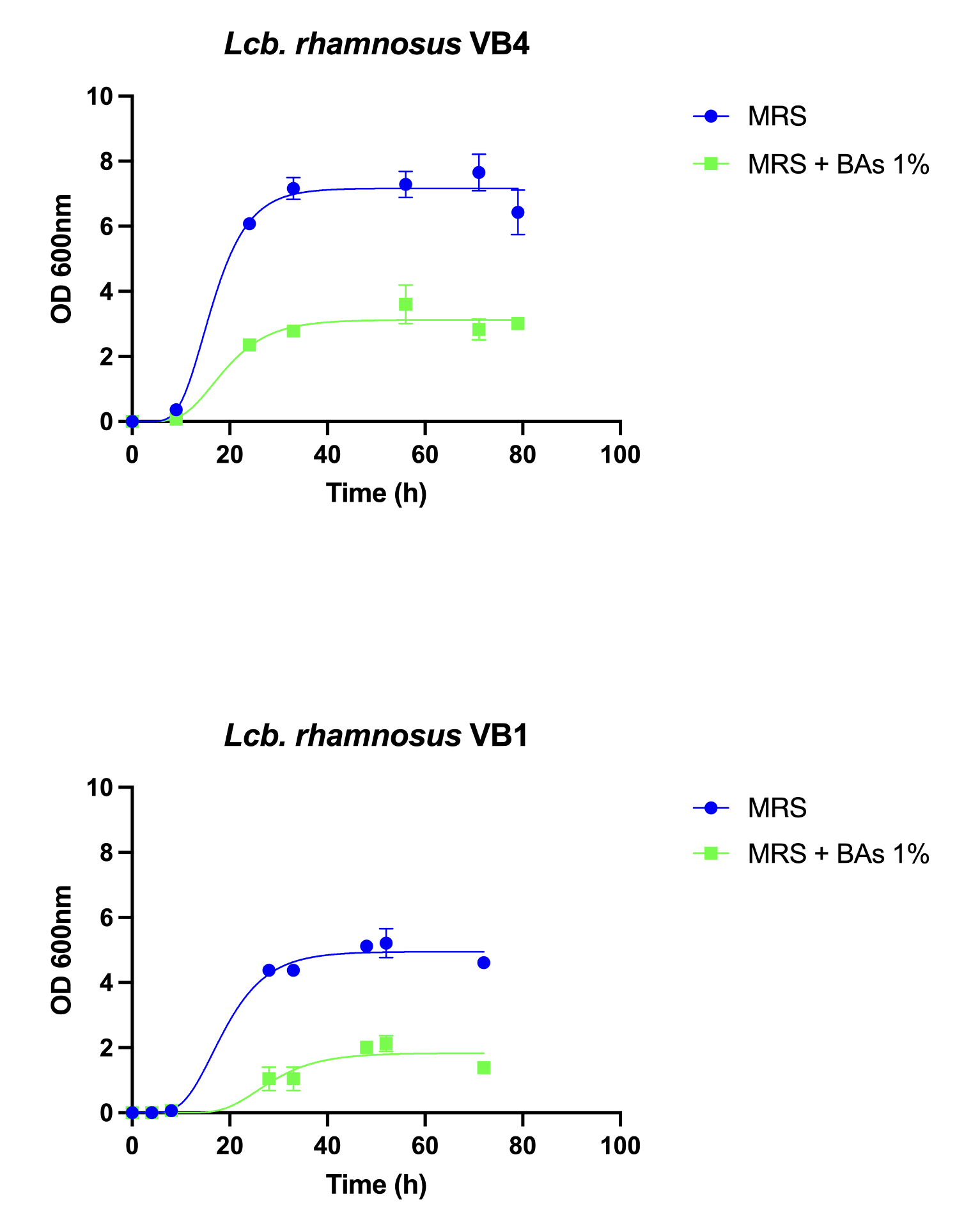

Supplement: Supplementary file 1 [file biomolecules-15-00086-s001.zip › Figure S4.tiff]

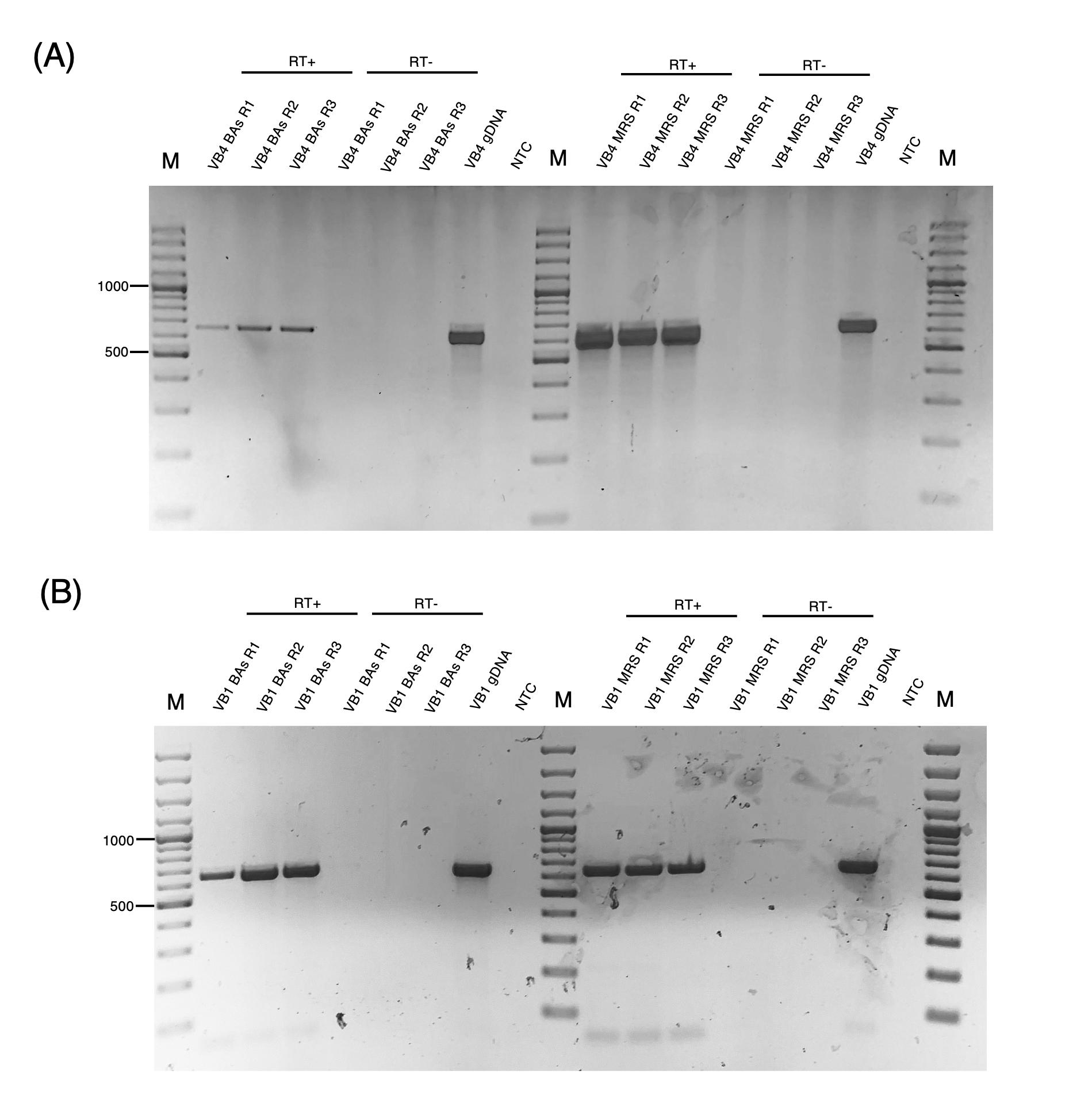

Supplement: Supplementary file 1 [file biomolecules-15-00086-s001.zip › Figure S5.tiff]
